# A High-Coverage Mesolithic Aurochs Genome and Effective Leveraging of Ancient Cattle Genomes Using Whole Genome Imputation

**DOI:** 10.1093/molbev/msae076

**Published:** 2024-04-25

**Authors:** Jolijn A M Erven, Amelie Scheu, Marta Pereira Verdugo, Lara Cassidy, Ningbo Chen, Birgit Gehlen, Martin Street, Ole Madsen, Victoria E Mullin

**Affiliations:** Groningen Institute of Archaeology, University of Groningen, Groningen, The Netherlands; Smurfit Institute of Genetics, Trinity College Dublin, Dublin D02 PN40, Ireland; Smurfit Institute of Genetics, Trinity College Dublin, Dublin D02 PN40, Ireland; Palaeogenetics Group, Institute of Organismic and Molecular Evolution (iOME), Johannes Gutenberg-University Mainz, 55099 Mainz, Germany; Smurfit Institute of Genetics, Trinity College Dublin, Dublin D02 PN40, Ireland; Smurfit Institute of Genetics, Trinity College Dublin, Dublin D02 PN40, Ireland; Key Laboratory of Animal Genetics, Breeding and Reproduction of Shaanxi Province, College of Animal Science and Technology, Northwest A&F University, Yangling 712100, China; Institute for Prehistory and Protohistory, University of Cologne, 50931 Cologne, Germany; LEIZA, Archaeological Research Centre and Museum for Human Behavioural Evolution, Schloss Monrepos, D - 56567 Neuwied, Germany; Animal Breeding and Genomics, Wageningen University and Research, Wageningen, The Netherlands; Smurfit Institute of Genetics, Trinity College Dublin, Dublin D02 PN40, Ireland

**Keywords:** cattle, imputation, aurochs, ancient genomics, domestication

## Abstract

Ancient genomic analyses are often restricted to utilizing pseudohaploid data due to low genome coverage. Leveraging low-coverage data by imputation to calculate phased diploid genotypes that enables haplotype-based interrogation and single nucleotide polymorphism (SNP) calling at unsequenced positions is highly desirable. This has not been investigated for ancient cattle genomes despite these being compelling subjects for archeological, evolutionary, and economic reasons. Here, we test this approach by sequencing a Mesolithic European aurochs (18.49×; 9,852 to 9,376 calBCE) and an Early Medieval European cow (18.69×; 427 to 580 calCE) and combine these with published individuals: two ancient and three modern. We downsample these genomes (0.25×, 0.5×, 1.0×, and 2.0×) and impute diploid genotypes, utilizing a reference panel of 171 published modern cattle genomes that we curated for 21.7 million (Mn) phased SNPs. We recover high densities of correct calls with an accuracy of >99.1% at variant sites for the lowest downsample depth of 0.25×, increasing to >99.5% for 2.0× (transversions only, minor allele frequency [MAF] ≥ 2.5%). The recovery of SNPs correlates with coverage; on average, 58% of sites are recovered for 0.25× increasing to 87% for 2.0×, utilizing an average of 3.5 million (Mn) transversions (MAF ≥2.5%), even in the aurochs, despite the highest temporal distance from the modern reference panel. Our imputed genomes behave similarly to directly called data in allele frequency-based analyses, for example consistently identifying runs of homozygosity >2 Mb, including a long homozygous region in the Mesolithic European aurochs.

## Introduction

Cattle are ubiquitous and have been a significant economic and cultural resource for millennia. Taurine cattle (*Bos taurus*) were initially domesticated from the now extinct aurochs (*Bos primigenius*) circa 10,500 years BP in southwest Asia (Vigne et al. 2017), with additional introgression from local wild populations in Africa, Europe, and the Levant (Park et al. 2015; Verdugo et al. 2019). Indicine cattle (*Bos indicus*) are adapted to warmer climates and descend from the recruitment of a divergent aurochs population (*Bos namadicus*), likely in the Indus Valley region ∼8,000 years BP (Patel and Meadow 2017). Cattle genetic variation is highly studied, but their evolutionary history is incompletely understood, with ancient genome investigation required to uncover key processes in prehistory. For example, human-mediated movement of indicine cattle resulted in widespread admixture between taurine and indicine cattle in southwest Asia at least 4,200 years BP, resulting in hybrid cattle that persist in the region today (Verdugo et al. 2019). However, as archeological remains are usually low in overall DNA concentration and endogenous DNA content, ancient genomes are typically low coverage (<5×; Daly et al. 2018; Frantz et al. 2019; Verdugo et al. 2019; Librado et al. 2021), preventing confident diploid calling and limiting analyses to pseudohaploid data, the sampling of one allele per variant site.

Genotype imputation—the statistical inference of unobserved genotypes by utilizing reference panels of haplotypes—is now a widely used methodological approach in ancient human genomics (Gamba et al. 2014; Martiniano et al. 2017; Cassidy et al. 2020). Specifically the development of GLIMPSE, a tool created for imputation from low-coverage genomes (Rubinacci et al. 2021), has enabled efficient imputation of large ancient human data sets (Hui et al. 2020; Clemente et al. 2021; Rohland et al. 2022; Sousa da Mota et al. 2023). Imputing low-coverage ancient genomes enables inferences of genome-wide diploid genotypes, diversifying analyses to include haplotype-focused or genealogical methods, e.g. inference of autozygosity within, and identity by descent (IBD) between genomes. While genotype imputation is regularly used in modern livestock genetics, including cattle (Hayes and Daetwyler 2019), and has been applied to ancient pigs and horses (Erven et al. 2022; Todd et al. 2023), the efficacy of imputation has not been explored in ancient cattle.

In order to explore the imputation and subsequent analyses of ancient *Bos*, we sequenced high-coverage genomes (>18×) of a Mesolithic German female aurochs and an Early Medieval Dutch cow. We combine these with two previously published high (>13×) coverage ancient genomes (a Neolithic Anatolian domesticate and a Medieval taurine × indicine hybrid from Iraq; Verdugo et al. 2019) and three modern cattle genomes of different ancestries (European *B. taurus*, African *B. taurus*, and *B. indicus*). A reference panel of 171 publicly available modern cattle genomes, composed of *B. taurus* and *B. indicus*, was aligned and curated for 21.7 million (Mn) single nucleotide polymorphisms (SNPs). By downsampling the test samples, imputing with GLIMPSE, and comparing analyses from direct genotype calls, we establish that this approach is both feasible and desirable for leveraging low-coverage ancient genome sequences, even for the extinct European aurochs.

## Results and Discussion

### The First High-Coverage Ancient European Aurochs and Domestic Cattle Genomes

**Fig. 1.**
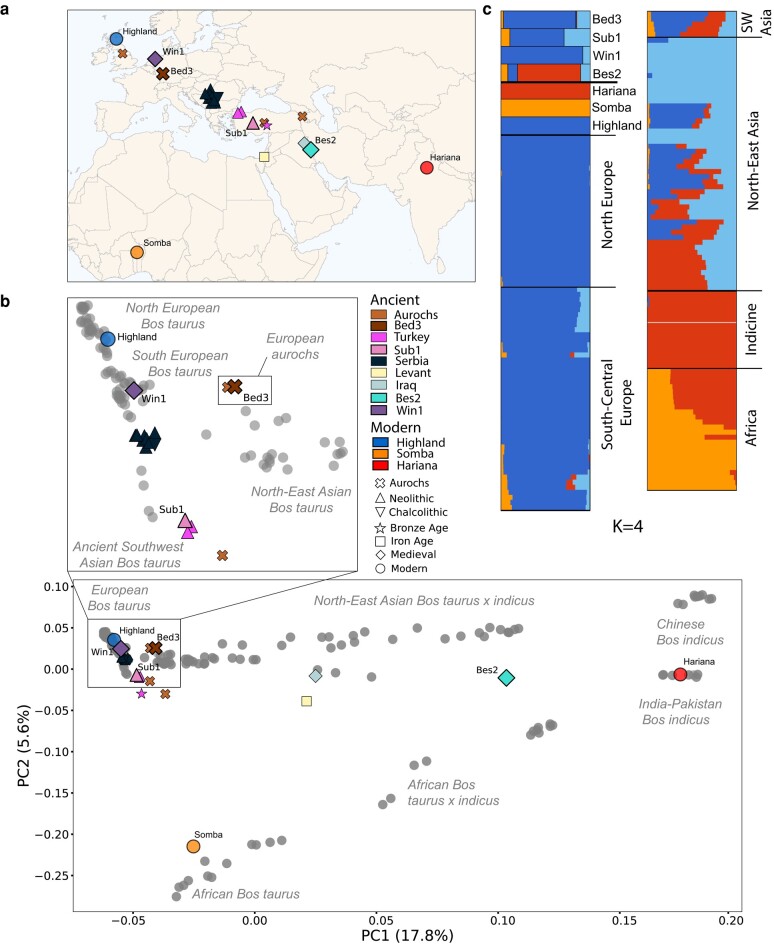
PCA and model-based clustering of ancient cattle. a) Geographical distribution of the samples projected in b). The four ancient genomes used to test imputation are labeled by sample name, and the three modern test individuals are labeled by breed. b) The new high-coverage genomes, Bed3 and Win1, are projected onto modern cattle diversity along with previously published ancient cattle and the three modern test samples. The test genomes are represented by diploid calls while previously published low-coverage ancient samples are pseudohaploid (supplementary table S1, Supplementary Material online). Test genomes are labeled as in a). Bed3 clusters with the previously published British aurochs CPC98, while Win1 clusters with modern Southern European *B. taurus*. c) Model-based clustering using ADMIXTURE of the test samples and modern cattle (*K* = 4). The imputation test samples are labeled as in a), and the comparative modern data set is arranged by geographic origin of breed.

Two ancient cattle genomes have been published with sequencing density sufficient for accurate genome-wide genotype calls: a Neolithic Turkish sample (Sub1; 6,221 to 6,024 calBCE, 13.85×) and a Medieval taurus–indicus hybrid sample from Iraq (Bes2; 1,295 to 1,398 calCE, 13.8×; Verdugo et al. 2019). Here, we additionally report the first ancient European domesticate to high coverage, a 18.69× Early Medieval Dutch female (Win1) from Winsum-Bruggeburen (424 to 569 calCE). We also report the first high-coverage Mesolithic female European aurochs (Bed3; 9,852 to 9,376 calBCE) 18.49× genome excavated in Bedburg-Königshoven, Germany (supplementary table S1, Supplementary Material online). When we coanalyze these two genomes with previously published data using standard allele frequency-based analyses (Fig. 1; supplementary fig. S1, Supplementary Material online), we demonstrate the well-established genetic variation of modern cattle, with distinct *B. taurus* and *B. indicus* populations (MacHugh et al. 1997; Bovine HapMap Consortium et al. 2009; Chen et al. 2018). Win1 clusters with modern southern and central European cattle, and Bed3 falls close to a published younger Mesolithic British aurochs (CPC98—5,746 to 5,484 calBCE; Park et al. 2015). As expected, Sub1 clusters with other Neolithic Anatolian samples, while the position of Bes2 confers its hybrid ancestry (Verdugo et al. 2019).

### Ancient Cattle Genomes Impute Nonrare Alleles to High Accuracy

We assembled, aligned, and variant called a phased reference panel of 171 published high-coverage (>7.6×) modern cattle genomes of varying ancestries (*B. taurus* and *B. indicus*) and with a geographical distribution including Europe, Africa, and Asia (Materials and Methods—Variant Discovery; supplementary table S2 and fig. S1, Supplementary Material online). Variants called with Graphtyper were curated with stringent filters (Materials and Methods—Variant Discovery), resulting in 21,656,052 high-confidence SNPs, including 6,521,311 transversions (supplementary table S2 and fig. S1, Supplementary Material online).

**Fig. 2.**
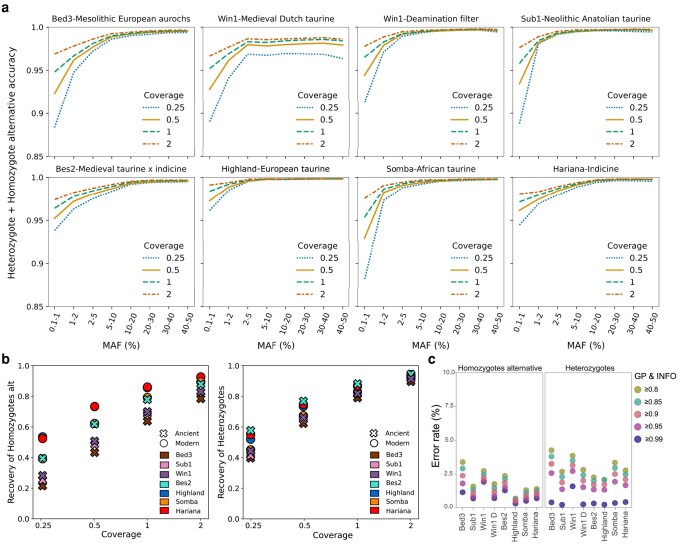
Accuracy, recovery, and error rate of imputation of heterozygote and homozygote alternative for the different downsampled coverages (MAF ≥ 2.5%, transversions only, GP ≥0.99, and INFO ≥0.99). a) Accuracy of imputation of heterozygote and homozygote alternative sites at different MAF bins and different downsampled coverages, transversions only, and with a GP and INFO filter ≥0.99. Downsampled coverage is denoted by line style and color. An additional graph is included for Win1 to demonstrate the positive effect on the accuracy of a deamination filter prior to imputation. b) Recovery rate of heterozygote and homozygote alternative for the different downsampled coverages (MAF ≥ 2.5%, transversions only, GP ≥0.99, and INFO ≥0.99), with samples denoted by shape and color. c) Error rates of the test samples shown for heterozygote and homozygote alternative for all test samples, including deamination filtered Win1, when imputed from 0.5× downsample (MAF ≥ 2.5%, transversions only) varying the quality thresholds.

The four ancient *Bos*, along with three previously published high-coverage (>28×) modern European *B. taurus* (Highland), African *B. taurus* (Somba), and Indian *B. indicus* (Hariana; Fig. 1), were successfully imputed from a range of downsampled coverages (0.25×, 0.5×, 1×, and 2×) using the newly created reference panel (Materials and Methods—Variant Discovery). Imputation accuracy, the concordance between imputed genotypes and high-quality validation genotypes for the high-coverage genomes was calculated for heterozygote and homozygote alternative sites via PICARD GenotypeConcordance (Toolkit 2019) at varying quality filters (supplementary figs. S2 and S3, Supplementary Material online). Here, we report the accuracy for our most stringent filters (genotype probability [GP] ≥0.99 and INFO ≥0.99), which results in the highest accuracy and lowest heterozygote error rate in our data (Fig. 2; supplementary fig. S3 and table S3, Supplementary Material online). This accuracy is relatively stable over the different genome coverages when tested with transversions only (Fig. 2a; supplementary table S4, Supplementary Material online). However, rare alleles are affected more by a reduction in genome coverage (Fig. 2a). Notably, this trend is also present at lower quality thresholds (supplementary fig. S3 and table S3, Supplementary Material online) and when all sites (transitions and transversions) are considered (supplementary fig. S4 and table S3, Supplementary Material online).

Across the coverage range, more common alleles have high accuracy, but this reduces for rarer alleles, with a marked falling off at minor allele frequency (MAF) < 5% (Fig. 2a). The lowest rare allele accuracies are observed in the Mesolithic European aurochs, Neolithic Anatolian domestic and modern African taurine samples. This reduction in accuracy is likely due to the underrepresentation of these ancestries in the reference panel, and a similar trend is also observed in non-European ancient humans (Sousa da Mota et al. 2023; Materials and Methods—Variant Discovery; Fig. 1; supplementary fig. S1 and table S2, Supplementary Material online). Indeed, the modern Highland test sample is well represented in the reference panel through the inclusion of individuals from the same breed and breeds of similar ancestry and shows a less severe reduction at rare alleles (MAF <5%). Simulated ancient human data have demonstrated that divergence from the reference panel could also be a factor in particular when imputing low-coverage data (Escobar-Rodríguez and Veeramah 2024). Additionally, heterozygote sites have a higher accuracy than homozygote alternative sites at more common (MAF >10%) alleles (supplementary tables S3 and S4, Supplementary Material online).

When we consider transversions with a MAF minimum threshold of 2.5%, the lowest accuracy is observed at the lowest downsample coverage of 0.25× (Win1 96.8%, Bed3 99.08%, Bes2 99.1%, and Sub1 99.52%; supplementary table S5, Supplementary Material online). It is interesting that, despite the highest temporal (>10,000 years) distance from the contemporary reference panel, the aurochs Bed3 performs well, for example better than the more recent European domesticate Win1. This implies that the modern reference imputation panel contains a substantial degree of segregating European wild haplotypes, presumably due to introgressions over the thousands of years when wild and herded animals cohabited on the continent (Park et al. 2015; Verdugo et al. 2019). A similar pattern has also been observed in the imputation of ancient humans (Martiniano et al. 2017; Sousa da Mota et al. 2023); for example, Indigenous Americans are accurately imputed despite the lack of unadmixed Indigenous American genomes in a reference panel (Sousa da Mota et al. 2023).

In our data, despite its relatively young provenance, Win1 has an elevated damage profile at CpG sites in the first and last 30 bp of the sequencing reads (supplementary fig. S5, Supplementary Material online) and the highest error rate for homozygote alternative and heterozygote sites (GP ≥0.99 and INFO ≥0.99; Fig. 2c; supplementary tables S3 to S5, Supplementary Material online). When we filter for potential deamination signals in this genome prior to imputation (Fig. 2a to c; supplementary fig. S6, Supplementary Material online; see Ancient Genome Alignment and Accuracy of Genotype Imputation), we demonstrate a decrease in error rate and an improvement in imputation accuracy at each downsampled coverage; at the lowest depth of 0.25×, accuracy increases from 96.8% to 99.6% for transversions with MAF ≥ 2.5%. While the deamination filter improved the accuracy of Win1, no substantial effect was demonstrated in the other ancient samples (supplementary fig. S7, Supplementary Material online).

Our ancient samples are of diverse provenance. In addition to the European wild and northwest European domestic genomes, we also successfully impute a Neolithic Anatolian *B. taurus* proximal to the origins of cattle in southwest Asia and a *B. indicus × B. taurus* hybrid from Iraq. These results suggest a wide potential for accurate genotype imputation of ancient cattle.

### Ancient Imputation Achieves Genome-Wide Genotype Recovery

Variation is demonstrated in the proportion of genotypes which are recovered, with a clear positive trend between genome coverage and site recovery rate; the recovery rate is determined by dividing the amount of correctly imputed variants by the total amount of variants in their high-quality counterparts (Fig. 2b; supplementary tables S3 to S5 and fig. S8, Supplementary Material online). The average recovery rate of heterozygote, homozygote alternative, and homozygote reference sites for the ancient genomes at a depth of 2× was 87.3% (3.0 Mn sites), while a rate of 58.0% (2.1 Mn sites) was achieved at a depth of 0.25× (MAF ≥2.5%, transversions only, GP ≥0.99, and INFO ≥0.99; supplementary table S5, Supplementary Material online). Across the downsampled coverages, the recovery rate for heterozygote and homozygote alternative sites was higher in the modern genomes (39.2% to 92.7% at 0.25× to 2×) than the ancients (30.6% to 91.2%; MAF ≥2.5%, transversions only; Fig. 2b). Additionally, when partitioning the data by MAF bins, the recovery rate differs between heterozygote and homozygote alternative to the reference genome, where heterozygotes have a higher recovery rate with more common alleles than homozygote alternative, a trend which reverses with rare alleles, mirroring the accuracy result. Taken together, this suggests a challenge in the imputation of heterozygote sites with low MAFs (supplementary tables S3 and S4, Supplementary Material online).

The recovery rate can be increased by reducing the GP and INFO filtering threshold (supplementary figs. S8 and S9, Supplementary Material online), particularly for homozygote alternative alleles (supplementary table S3, Supplementary Material online). However, this increase is accompanied by a reduction in imputation accuracy and an increase in error rate (Fig. 2; supplementary figs. S2 and S3 and table S3, Supplementary Material online). The tradeoff between recovery and accuracy should be driven by the subsequent analysis requirements; haplotype-aware methods, such as IBD, require a stringent GP threshold (Ariano et al. 2022; Ringbauer et al. 2024) and here accuracy should be prioritized.

### A Sliding Window Analysis Demonstrates Variations in Imputation Accuracy and Recovery

We explored imputation accuracy and recovery across the genome by using a sliding window approach (500 kb window, 100 kb step) and including homozygote reference, homozygote alternative, and heterozygote sites for 0.5× imputed genotypes (MAF ≥2.5%, transversions only, GP ≥0.99, and INFO ≥0.99). Accuracy and recovery across the genomes are relatively stable in all test genomes (supplementary figs. S10 to S16, Supplementary Material online). We considered the bottom 0.01 percentile of sliding windows as outlier regions of reduced recovery and accuracy (supplementary tables S6 to S8, Supplementary Material online).

Fourteen outlier regions of reduced accuracy were identified in four or more test samples, and 12 of these are also reduced recovery regions (supplementary table S6, Supplementary Material online). Only 2 of these 14 are exclusive to the 4 ancient samples in this study. Interrogation of these demonstrates that the majority of these regions contain olfactory receptor and immunity-related genes, regions of CNVs, and tandem repeats (supplementary table S6, Supplementary Material online). Furthermore, eight of these have previously been identified as difficult to impute in modern taurine cattle (Zhang et al. 2023) including one of the two ancient only regions in this study. Additionally, we identify 30 regions of reduced recovery but not reduced accuracy that are present in four or more test samples (supplementary table S7, Supplementary Material online), with only five exclusive to the four ancient samples. Seventeen of these 30 regions are within or overlap with the first and last 1 Mb of chromosomes. Taken together, this suggests these regional inaccuracies are due to genome architecture.

### Imputed Genomes Allow Accurate Analysis Outcomes

**Fig. 3.**
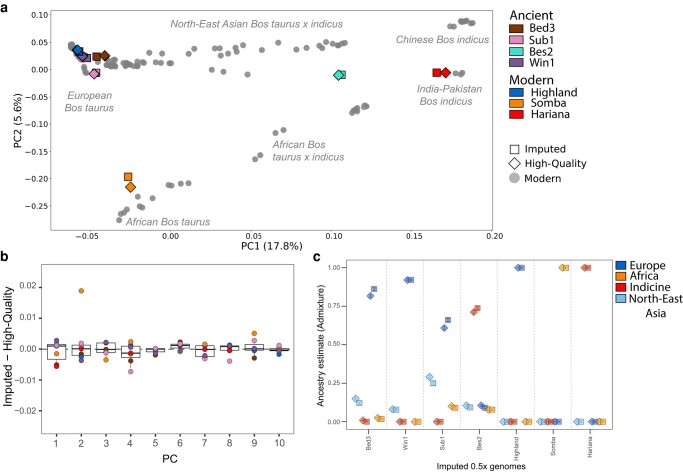
PCA and ADMIXTURE of imputed and high-quality genotypes of the seven test samples onto the modern cattle reference panel. a) Projections for 0.5× imputed and high-quality diploid genotypes of the test samples onto the modern reference panel along the first two eigenvectors. Data set was filtered for a MAF ≥ 2.5%, transversions, and LD pruning resulting in 812,801 SNPs. Test samples are denoted by color with imputed and high-quality represented by squares and diamonds, respectively, while the reference panel individuals are plotted as circles. b) Boxplots of the normalized differences in the coordinates of the high-quality and 0.5× imputed genomes for the first ten principle components. The horizontal lines of the boxplot represent the first quartile, median, and third quartile; whiskers represent 1.5 times the quartile range. c) Comparison of the ancestral components modelled by ADMIXTURE (K = 4) based on 800,694 SNPs (MAF ≥ 2.5%, transversions and LD pruning) run with the reference panel and the seven test samples. Here we display results for the 0.5x imputed (squares) and high-quality validation (diamonds) genotypes. Error bars represent the deviation from the mean measured as standard errors calculated from 1,000 replicates.

We conducted unsupervised frequency-based analyses, which are commonly used in ancient population genomics (i.e. principal component analysis [PCA] and ADMIXTURE) demonstrating a positive trend between accuracy and increasing downsample coverage (supplementary figs. S17 to S21, Supplementary Material online). For example, the eigenvector difference between the high-quality and imputed replicate in the projection PCA decreases with increasing downsample coverage (Fig. 3; supplementary fig. S17, Supplementary Material online). Here, we discuss analyses utilizing the 0.5× downsample imputation (GP ≥0.99 and INFO ≥0.99; MAF >0.025 and transversions only; Fig. 3). The projection PCA demonstrates close clustering between each high-quality and imputed replicate, with the greatest eigenvector difference observed for the modern African taurine individual (Somba) across PC2 (Fig. 3). ADMIXTURE analysis estimated similar spectra of ancestral component profiles between imputed and high-quality genotypes (Fig. 3c; supplementary figs. S18 to S21, Supplementary Material online). These successful replications demonstrate consistency between our imputed and high-quality data sets, including the analysis of the most temporally distant sample, the ∼11,500-year-old Mesolithic European aurochs (Bed3).

**Fig. 4.**
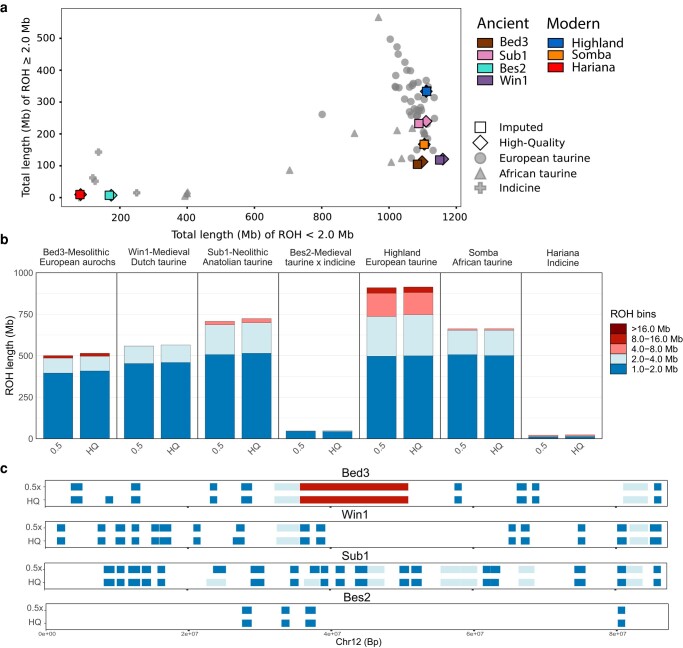
ROH estimates for the 0.5× imputed and the high-quality genotypes. a) The total length of ROH ≥ 2 Mb and ROH < 2 Mb (includes ≥0.5 Mb) plotted with a subset of the reference panel as a background. Filtering included MAF ≥2.5%, transversions only, and no missingness. b) The total length of ROH split into length bins for the seven test samples. The number of sites used for analysis = 481,786 filtered for a MAF ≥2.5%, transversions only, and no missingness. c) ROH along Chr12 for the four ancient genomes at 0.5×, and high-quality demonstrating the general consistency between the imputed and high-quality data set; filters are the same as b).

For the first time, we applied a runs of homozygosity (ROH) analysis on ancient cattle and compared high-quality and imputed data (Fig. 4). Patterns of ROH were consistent when comparing the 0.5× downsampled imputation to the high-quality genotypes (Fig. 4; supplementary figs. S22 to S25, Supplementary Material online). This was true for both genome-wide summaries of ROH (Fig. 4a and b) and genome-wide distribution of ROH (supplementary figs. S22 to S25, Supplementary Material online). In particular, a large ROH of 15.8 Mb on chromosome 12 of the Mesolithic aurochs is successfully identified using both imputed and high-quality genotypes (Fig. 4c; supplementary fig. S22, Supplementary Material online).

Moreover, imputation seems robust for estimating both recent genealogical and deeper population histories via alternately large and small ROH (<2 Mb; Fig. 4a). In the test samples, the most pronounced inbreeding is in the modern Highland sample; a feature typical of European production breeds (Fig. 4; supplementary fig. S26, Supplementary Material online; Purfield et al. 2012) and a pattern absent from the ∼1.5 kyr Medieval Dutch sample. The Medieval zebu hybrid, Bes2, had markedly low levels of ROH, reflecting the large effective population size and genome diversity that is a well-established feature of *B. indicus* history (Bovine HapMap Consortium et al. 2009; Murray et al. 2010; Chen et al. 2018).

We demonstrate that imputation of ancient cattle, including of the extinct European aurochs, is a feasible methodology for future studies. The success of the imputation of aurochs implies segregating haplotypes in the modern reference panel, most likely from introgression. While damage is potentially disruptive, this is correctable with a deamination aware approach. Imputation accuracy is high and is relatively consistent across the downsampled coverages, demonstrating the feasibility of imputing ancient genomes as low as 0.5× or even lower. This is demonstrated through the consistency in the analysis between imputed and high-quality genotypes. The successful imputation of ancient cattle presents the opportunity for haplotype-aware analysis in the future.

## Materials and Methods

### Ancient Processing—Bed3 and Win1

The two archeological samples were processed in dedicated ancient DNA laboratories in Trinity College Dublin (Win1) and Johannes Gutenberg University of Mainz (Bed3).

#### Win1

A petrous bone, GIA collection number 3848, was excavated in 1997 from the site Winsum-Bruggeburen in The Netherlands. Site occupation was from 7th century BCE to 14th century CE and is thought to have been a military Roman outpost (Bos et al. 1997).

Sample preparation of Win1 was performed as described in Verdugo et al. (2019). In brief, a wedge of bone was drilled and subsequently powdered using a mixer mill. DNA extraction of ∼150 mg bone powder followed a 48-h two-step double extraction of two 24-h digestion steps (37 °C) with fresh extraction buffer (0.5 M EDTA pH 8, 1 M Tris-HCl, 2% sodium dodecyl sulfate, and 100 μg/mL proteinase K) added after the first 24-h period. Digestion was followed by a Tris-EDTA wash using Amicon Ultra 4 mL filter (Merck Millipore) followed by a DNA purification step using the “QIAQuick minElute purification kit” (Qiagen) and eluted in 40 μL of EB buffer + Tween 20 (Sigma-Aldrich; Mattiangeli 2023). Prior to library preparation, a UDG treatment was performed using 16.25 µL of purified DNA and 5 μL (1 U/1 µL) USER enzyme (New England BioLabs, Inc.) and an incubation of 3 h at 37 °C. Double-stranded libraries were prepared (Meyer and Kircher 2010) and sequenced (100 bp SE) on an Illumina HiSeq 2000 at Macrogen, Inc. (1002, 254 Beotkkot-ro, Geumcheon-gu, Seoul 153-781, Republic of Korea).

Radiocarbon dating was performed at 14CHRONO at Queen's University Belfast (UBA-29049: 1,556 ± 31 BP) and calibrated dates produced by OxCal 4.4 IntCal20 atmospheric curve (Ramsey 2009; Reimer et al. 2020; supplementary fig. S28, Supplementary Material online).

#### Bed3

A petrous from an aurochs skull, find number 104/102-1, was excavated from the Mesolithic site of Bedburg-Königshoven in Germany (Street 1999) along with numerous animal bones and two red deer skull headdresses (Wild et al. 2020). The site—today destroyed by lignite mining—was a dump area, formerly located at the bank of a prehistoric wetland close to the former river Erft (Street 1999, 2020).

Sample preparation of Bed3 was performed as described for sample CTC in Botigué et al. (2017) and Ch22 and Th7 in Verdugo et al. (2019), with the following alterations: two independent extractions of 1 g of bone powder were performed with a prelysis EDTA wash for 30 min at room temperature. Extracts underwent UDG treatment, and subsequently, 15 double-stranded sequencing libraries (Meyer and Kircher 2010) were prepared and sequenced (100 bp SE) on an Illumina HiSeq 2000 at Macrogen, Inc. (1002, 254 Beotkkot-ro, Geumcheon-gu, Seoul 153-781, Republic of Korea).

Radiocarbon dating was performed by CologneAMS at the University of Cologne in 2014 as part of the Mesolithic project D4 of the CRC 806 (COL-2680-2.1: 10,036 ± 42) and calibrated dates produced by OxCal 4.4 IntCal20 atmospheric curve (Ramsey 2009; Reimer et al. 2020; supplementary fig. S27, Supplementary Material online).

### Ancient Genome Alignment

Fastq files were processed through a pipeline similar to Verdugo et al. (2019). Reads were trimmed for adapter sequences using cutadapt (v. 1.1; Martin 2011; *-0 1 & -m 30*) and aligned to ARS-UCD1.2 with the addition of the Y from BosTau5 using the Burrows-Wheeler Alignment (v. 0.7.5a-r405; Li and Durbin 2009) with the subcommand aln (*-l 1024 -n 0.01 -o 2*). BAM files were sorted with SAMtools (v. 1.9; Li et al. 2009) and duplicates removed with Picard (v. 2.20.3; Toolkit 2019). Indel realignment was performed using the Genome Analysis Toolkit (v. 3.3.0; McKenna et al. 2010), SAMtools implemented for mapping quality filtering (*-q25*), and coverage calculations performed by Qualimap (v. 2.1.3; Okonechnikov et al. 2016). To further minimize the effects of deamination, the soft clipping of five base pairs at both ends of the reads was performed. As DNA extracts were subject to UDG treatment, deamination-derived damage patterns typical of aDNA damage were calculated with PMDtools (Skoglund et al. 2014) separating deamination-derived damage patterns in CpG and non-CpG sites with the parameters *–playtypus –requirebaseseq 30*.

### Modern Genome Processing

Publically available fastq files of 201 individuals (supplementary table S9, Supplementary Material online) were downloaded and processed through the following pipeline. Reads were trimmed for adapters using Trimmomatic (v 0.39) and aligned with BWA mem (v. 07.13; Li 2013). Reads were sorted and duplicates removed (PICARD 2.20.3; Toolkit 2019) and properly paired reads retained (SAMtools v 1.9; Li et al. 2009). Indel alignment was performed (GATK version v. 3.3.0; McKenna et al. 2010) and reads filtered via samtools for mapping quality (q 25).

### Variant Discovery

Variants (SNPs and insertions or deletions [INDELs]) were called from 201 mapped and filtered bam files of modern cattle (supplementary table S9, Supplementary Material online) with an average coverage above 7.6× using Graphtyper (Eggertsson et al. 2017), running each chromosome in parallel (Version 2.7.4). SNPs were removed if they were within 3 bp of another INDEL or SNP with vcftools 0.1.17. INDELs were removed, and SNPs were filtered for biallelic alleles, a minimum genotype depth of 6×, a maximum genotype depth of three times the average genomic coverage of that individual, a minimum quality of 25, and a minimum genotype quality of 20 with vcftools version 0.1.17. SNPs were further filtered according to Graphtyper's guidelines with bcftools filter version 1.12 (*QD > 2.0*, *SB < 0.8*, *MQ > 40.0*, *LOGF > 0.5*, *AAScore > 0.5*; Li 2011). As a final filtering step singletons, repetitive regions and genotypes with more than 20% missingness were removed with vcftools version 0.1.17 (Danecek et al. 2011), resulting in 21,656,053 high-quality SNPs (supplementary table S2, Supplementary Material online). After filtering, individuals with more than 20% missingness and individuals with second-degree relatives (>0.0885 score, vcftools relatedness2) were removed from the data set resulting in 171 individuals (supplementary tables S2 and S9, Supplementary Material online). The reference panel consists of 75 European *B. taurus*, 27 Asian *B. taurus*, 15 African *B. taurus*, 10 African *B. taurus* × *B. indicus*, 5 southwest Asian B. taurus × *B. indicus*, 23 North East Asian *B. taurus* × *B. indicus*, and 16 *B. indicus*. The reference panel was phased using Beagle5 (Browning and Browning 2007), with the parameters *impute=false, window=40, overlap=4, gp=true ne=20000*.

### Pseudohaploid Data Set

The previously published ancient low- to medium-coverage genomes (0.1 to 3.8× coverage range; supplementary table S1, Supplementary Material online) were pseudohaploidized using ANGSD version 0.938 (Korneliussen et al. 2014) doHaploCall, with the following parameters: *doHaploCall* 1, *doCounts* 1, *dumpCounts* 1, minimum base quality of 30 (*-minQ 30*), minimum mapping quality of 25 (*-minMapQ 25*), retain only uniquely mapped reads (*-UniqueOnly 1*), remove reads flagged as bad (*-remove_bads*), remove triallelic sites (*-rmTriallelic 1e—4*), downscale mapping quality of reads with excessive mismatches (*-C 50*), discard 5 bases of both ends of the read (*-trim 5*), and remove transitions (-rmTrans 1). The abovementioned sites in the modern reference panel (21,656,053 high-quality SNPs) were used as input for ANGSD using the parameter *–sites*. As a sanity check, the major/minor alleles of the low-coverage ancient were compared to the modern reference panel and were removed if there were any discrepancies. ANGSD haplo files were transformed to plink tped files with the haploToPlink function from ANGSD version 0.938 and recoded into ped files with PLINK v.1.90 (Chang et al. 2015). Transitions were removed because transitions, unlike transversions, are most affected by postmortem deamination of DNA, which might increase the number of wrongly called SNPs. The restriction to transversions only is a standard approach in ancient DNA studies.

### Genotype Imputation

Four ancient (13.8 to 18.7× coverage range) and three modern genomes (28.4 to 32.7× range) were downsampled to 0.25×, 0.5×, 1.0×, and 2.0× on a chromosomal level using picard 2.20.0. The three high-coverage (>28×) modern cattle were selected to represent European *B. taurus* (Highland—ERR3305587), African *B. taurus* (Somba—ERR3305591), and Indian *B. indicus* (Hariana—SRS3120723). The downsampling was performed on chromosomal level so that average genomic coverage would not skew the downsampling process. Genotype likelihoods were computed for the downsampled and the original high-coverage genomes for the high-quality 21,656,053 SNPs mentioned in the variant calling section.

Genotype calls and likelihoods were generated according to the GLIMPSE version 1.1.1 pipeline (Rubinacci et al. 2021), with the command bcftools mpileup (version 1.12) with parameters *-I, -E, -a “FORMAT/DP,FORMAT/AD,INFO/AD*”, the reference genome and the abovementioned sites in the reference panel (-*T*) followed by bcftools call with the parameters *-Aim -C alleles*, and the abovementioned sites (-*T*). This step was performed on both downsampled and high-coverage genomes. The high-coverage genotype likelihoods were further filtered for a minimum base quality of 30, a minimum genotype quality of 25, a minimum genotype coverage of 8, a maximum genotype coverage of 3 times the average genomic coverage, and a minimum allelic balance of 40%, obtaining the validation gold standard genotype likelihoods.

Imputation was performed on the downsampled genomes using GLIMPSE v1.1.1 (Rubinacci et al. 2021), according to the GLIMPSE pipeline. Chromosomes were split into chunks of 2 Mb with a 200 kb buffer window with *GLIMPSE_chunk*. Imputation was performed on these chunks with default parameters using *GLIMPSE_phase* with the reference panel created in the section variant calling. The imputed chunks were ligated using *GLIMPSE_ligate* with default parameters. For imputation of the three modern test samples, reference panels without the test individual were created and subsequently used for imputation of the respective test sample.

The imputed data were filtered to keep only the most confidently imputed SNPs; this was done to decrease the chance of erroneous SNPs in our date, which can influence haplotype-based analyses such as IBD; this was done by filtering for a strict GP ≥ 0.99 and an INFO score ≥0.99; each sample was imputed and filtered separately. INFO score filtering was included as it improved heterozygote accuracy, especially for rare alleles and low downsampled coverages, in our data (supplementary fig. S2, Supplementary Material online). To further explore the effect of filtering on imputation accuracy and recovery, we varied the GP and INFO filters (0.8, 0.85, 0.9, 0.95, and 0.99).

### Accuracy of Genotype Imputation

Imputation accuracy, seen as genotype concordance between the imputed and the high-quality validation genotypes, was calculated with Picard GenotypeConcordance version 2.20.0 (Toolkit 2019). Imputation accuracy was calculated for heterozygotes, homozygote alternative (relative to the reference genome), and the combined alternative alleles; this was done for eight MAF bins and a MAF threshold. Picards' GenotypeConcordance tool was used with the high-quality validation genotypes as the *TRUTH_VCF*, the imputed genotypes as the *CALL_VCF*, and for the specific MAF bins mentioned previously with the *INTERVALS* parameter. Genotype concordance, called sensitivity in Picards' output, is calculated as TP/(TP+FN), where TP (true positives) stands for variants where the CALL matches the TRUTH, and FN (false negatives) stands for when variants do not match the reference. Recovery of genotypes is calculated as (TP+FN)/TotalHQ, where Total HQ stands for the genotypes present in the high-quality validation TRUTH.

Error rates were calculated by dividing the amount of incorrectly imputed genotypes, seen as different from the TRUTH genotype, by the total number of genotypes; this was calculated separately for heterozygote and homozygote alternative. Nonreference discordance (NRD), a measurement of error rate, was calculated following the formula published by Sousa da Mota et al. (2023), and NRD does not take correctly imputed homozygote reference sites into account, giving more weight to imputation errors at alternative sites.

Win1, a European taurine animal, demonstrates the lowest imputation accuracy of heterozygote and homozygote alternative alleles (Fig. 2). While all ancient samples underwent UDG treatment, deamination can still be detected at CpG sites due to the impacts of methylation (Gokhman et al. 2014), and in Win1, this is elevated throughout the first and last 30 bp of the sequencing reads (supplementary fig. S5, Supplementary Material online). We find that in this sample, the imputation accuracy can be improved when we filter for potential deamination signals prior to imputation. This was achieved by (i) setting heterozygote or homozygote reference genotypes as missing if the reference allele was T or A and the alternate allele was C or G, respectively, and (ii) setting heterozygote or homozygote reference genotypes as missing if the reference was T or A and the alternate allele was C or G, respectively. Using these filtered genotypes, we demonstrate an improvement in post imputation accuracy (supplementary figs. S2 and S3, Supplementary Material online).

Imputation accuracy and recovery throughout the genome were calculated in sliding windows of 500 kb with a step size of 100 kb; sliding windows were created with BEDTools (Quinlan and Hall 2010) makewindows. Imputation accuracy was calculated as the number of correctly imputed genotypes (heterozygotes, homozygote alternative, and homozygote reference), seen as similar to the TRUTH genotypes, divided by the total number of genotypes in the sliding window; sliding windows consisting of correctly imputed and total number of genotypes were created with BEDTools map, using the sliding windows (500 kb with a step of 100 kb) created earlier. This approach was repeated for the recovery, which was calculated by dividing the number of imputed genotypes, present in the TRUTH, by the total number of variants in the reference panel; this was done for each sliding window. The lowest imputation accuracy and recovery regions were extracted by filtering for the bottom 0.01 percentile, and these outlier regions were then merged with BEDTools merge. Genes in outlier regions were determined using RefSeq annotation NCBI Bostau 9 release, downloaded from UCSC genome browser (last accessed 20-03-2024), using BEDTools closest. To determine regions that were in the majority of the samples, BEDTools merge was used on the individual bed files, regions that overlapped were counted, with the parameter *-o sum*, and these regions were subsequently filtered to only be present in the majority of the samples (4). Unique regions in the test samples were determined by using BEDTools subtract.

### Downstream Analyses

The filtered imputed and high-quality genotypes were merged with the modern reference panel using PLINK v.1.90 (Chang et al. 2015). In the case of PCA and admixture analyses, the data were filtered for MAF >2.5%, transversions only, and linkage disequilibrium (indep-pairwise 50 5 0.5), resulting in 812,801 SNPs. For the ROH analysis, a subset of modern individuals <5% missingness (*N* = 60) was created and merged with the imputed genotypes. The filters on the ROH data set consisted of MAF >2.5%, no genotype missingness, and transversions only, resulting in 481,786 SNPs.

#### PCA including Ancient Pseudohaploid

The pseudohaploid ancient samples were merged with the data set containing the imputed and high-quality genotypes and modern reference panel, filtered for MAF >2.5%, transversions only, and linkage disequilibrium (indep-pairwise 50 5 0.5), resulting in 812,801 SNPs. Smartpca version 16000 was used to perform a PCA with default parameters (Patterson et al. 2006; Price et al. 2006). The first ten principal components were calculated using the modern reference panel; the pseudohaploid, imputed, and high-quality genotypes were projected (*lsqproject:yes*).

#### PCA to Test Imputation Genotypes

Smartpca version 16000 was used to perform a PCA with default parameters (Patterson et al. 2006; Price et al. 2006). The first ten principal components were calculated using the modern reference panel, and both the imputed and high-quality genotypes were projected (*lsqproject:yes*). PCA was performed on each imputed coverage separately.

#### Model-Based Admixture

ADMIXTURE v1.3.0 (Alexander et al. 2009) was used to estimate ancestry proportions for the modern reference panel, high-quality, and imputed genotypes. ADMIXTURE was ran for K between two and ten; for the best K's (four to five), a bootstrap with 1,000 replicates (−B 1000) was run to obtain the standard error and bias of admixture estimates. Admixture was ran on each imputed coverage separately.

#### ROH

ROHs were estimated with PLINK v1.90 (Chang et al. 2015) with the parameters *--homozyg --homozyg-density 50 --homozyg-gap 100 --homozyg-kb 500 --homozyg-snp 50 --homozyg-window-het 1 --homozyg-window-snp 50 --homozyg-window-threshold 0.05*, according to earlier studies (Gamba et al. 2014; Cassidy et al. 2016; Martiniano et al. 2017; Sousa da Mota et al. 2023). ROHs were estimated using a subset of moderns (<5% missingness), high-quality, and imputed genotypes for each imputed coverage separately.

## Supplementary Material

msae076_Supplementary_Data

## Data Availability

The raw reads for the two new ancient genomes will be deposited with ENA PRJEB74338. The accession numbers for the publicly available genomes used in the reference panel are noted in supplementary tables S1, S2, and S9, Supplementary Material online. The VCF of the phased reference panel will be made publicly available on Zenodo with the DOI 10.5281/zenodo.10912801.
