# Supplementary material for: A High-Coverage Mesolithic Aurochs Genome and Effective Leveraging of Ancient Cattle Genomes Using Whole Genome Imputation
Source: Mol Biol Evol. 2024 Apr 25;41(5):msae076. doi: 10.1093/molbev/msae076 (PMC11090068; doi:10.1093/molbev/msae076)
Supplement: msae076_Supplementary_Data [file msae076_supplementary_data.zip › SI_Figures_Cattle_Imputation_Resubmission.pdf]

Supplementary Figures for

A high coverage Mesolithic aurochs genome and effective leveraging of ancient cattle genomes using whole genome imputation.

Jolijn A.M Erven, Amelie Scheu, Marta Pereira Verdugo, Lara Cassidy, Ningbo Chen, Birgit Gehlen, Martin Street, Ole Madsen, Victoria E Mullin*

*corresponding author: mullinve@tcd.ie

Includes Figures S1 to S28

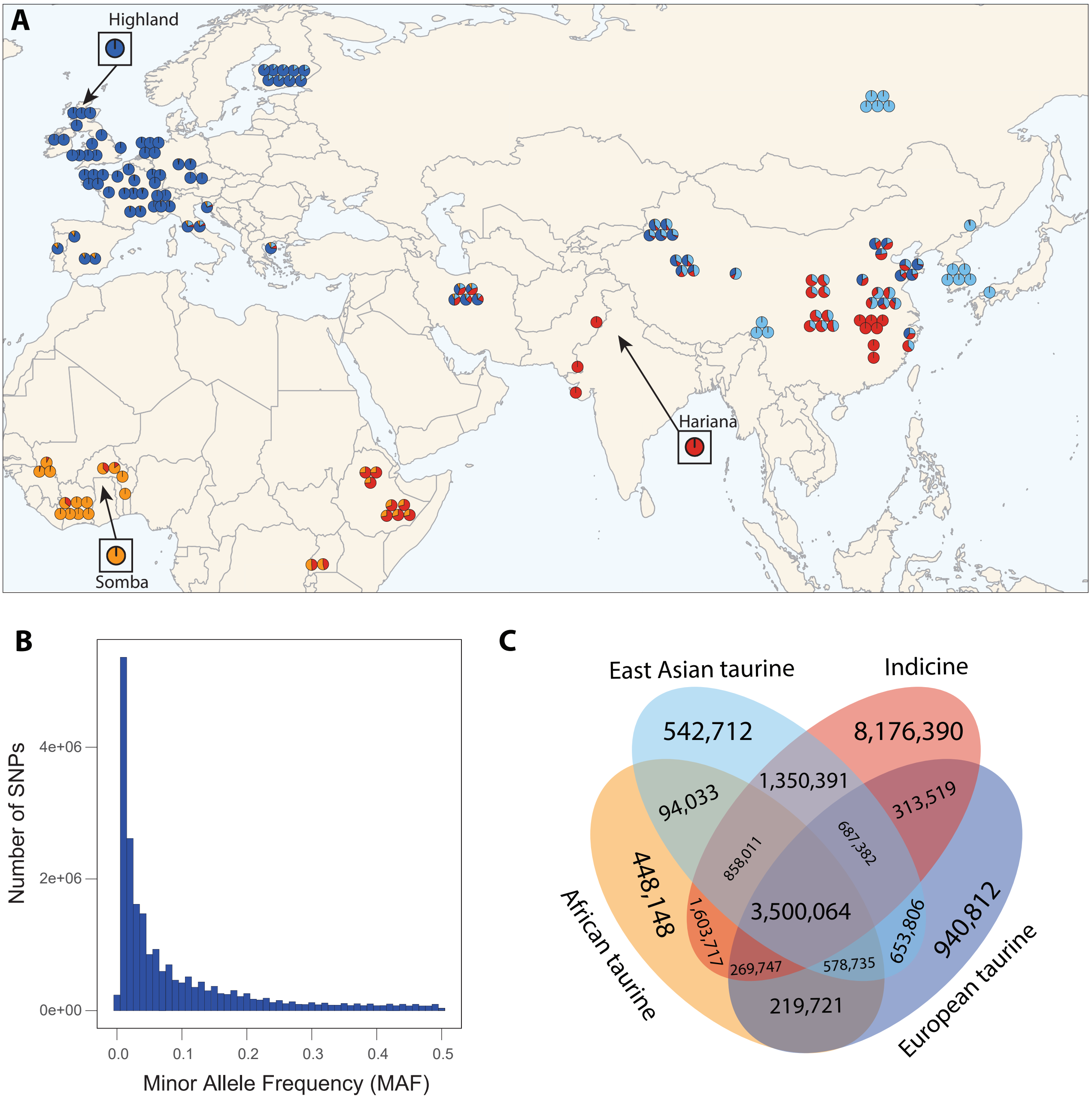

Figure S1. Geographical distribution of ancestries, Minor Allele Frequency (MAF) and shared and unique variants in the reference panel. A) Geographical distribution of the samples included in the reference panel. Colors denote European taurine (dark blue), African taurine (orange), North-East Asian taurine (light blue) and indicine (red). B) MAF distribution in the reference panel. C) Unique and shared variants of the four main ancestries in the reference panel, colors denote European taurine (dark blue), African taurine (orange), North-East Asian taurine (light blue) and indicine (red).

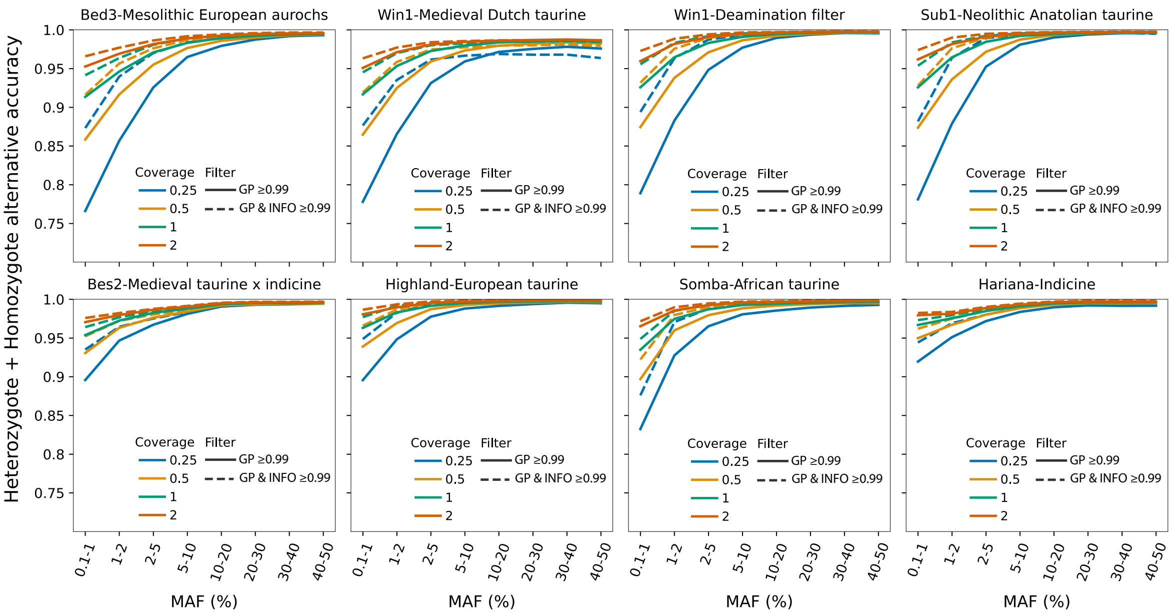

Figure S2. Accuracy of imputation of heterozygote and homozygote alternative sites at different minor allele frequency (MAF) bins and different GP and INFO filters. GP & INFO and GP filter are denoted by line style and colour. An additional graph is included for Win1 to demonstrate the positive effect on the accuracy of a deamination filter prior to imputation.

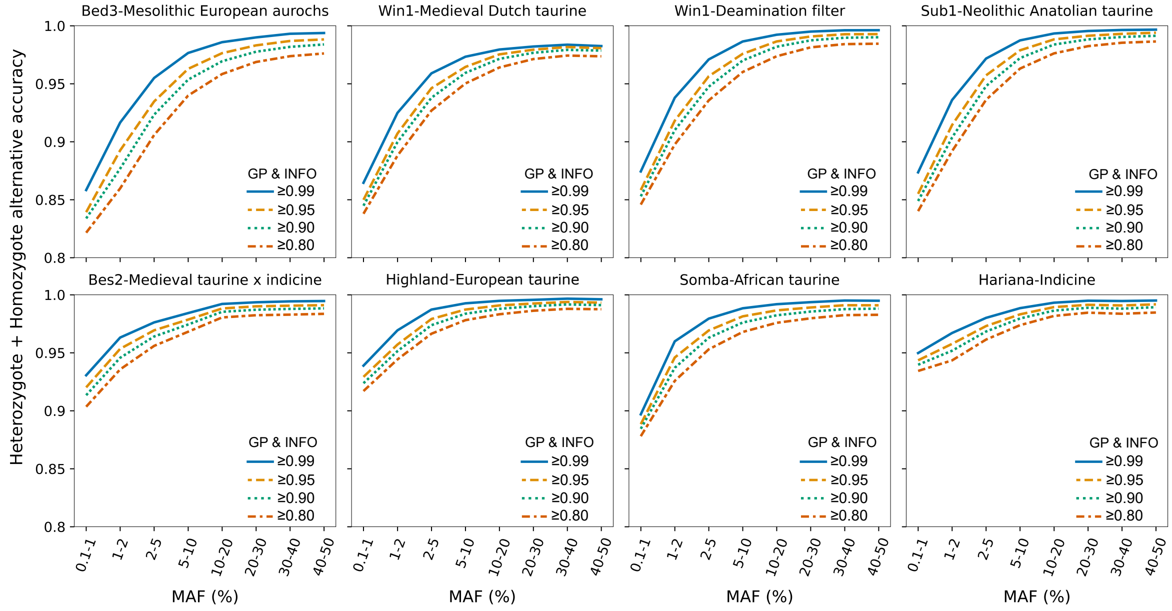

Figure S3. Accuracy of imputation of heterozygote and homozygote alternative sites at different minor allele frequency (MAF) bins and different GP and INFO filters, shown for 0.5x coverage for all sites. GP & INFO filter is denoted by line style and colour. An additional graph is included for Win1 to demonstrate the positive effect on the accuracy of a deamination filter prior to imputation.

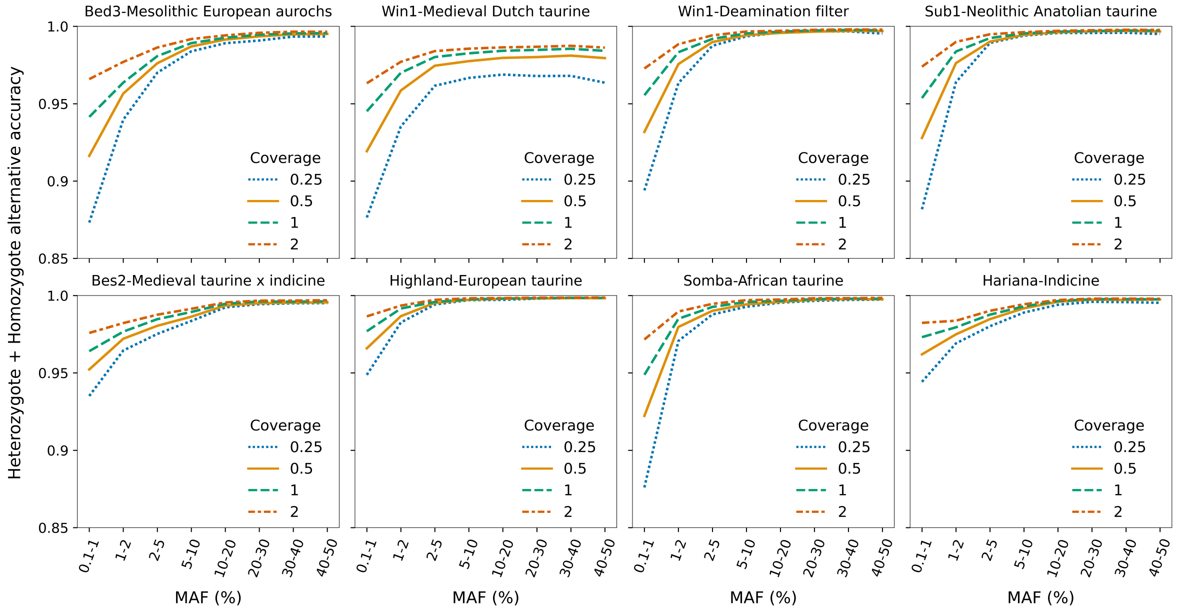

Figure S4. Accuracy of imputation of heterozygote and homozygote alternative sites at different minor allele frequency (MAF) bins and different downsampled coverages, for all sites. Downsampled coverage is denoted by line style and colour. An additional graph is included for Win1 to demonstrate the positive effect on the accuracy of a deamination filter prior to imputation.

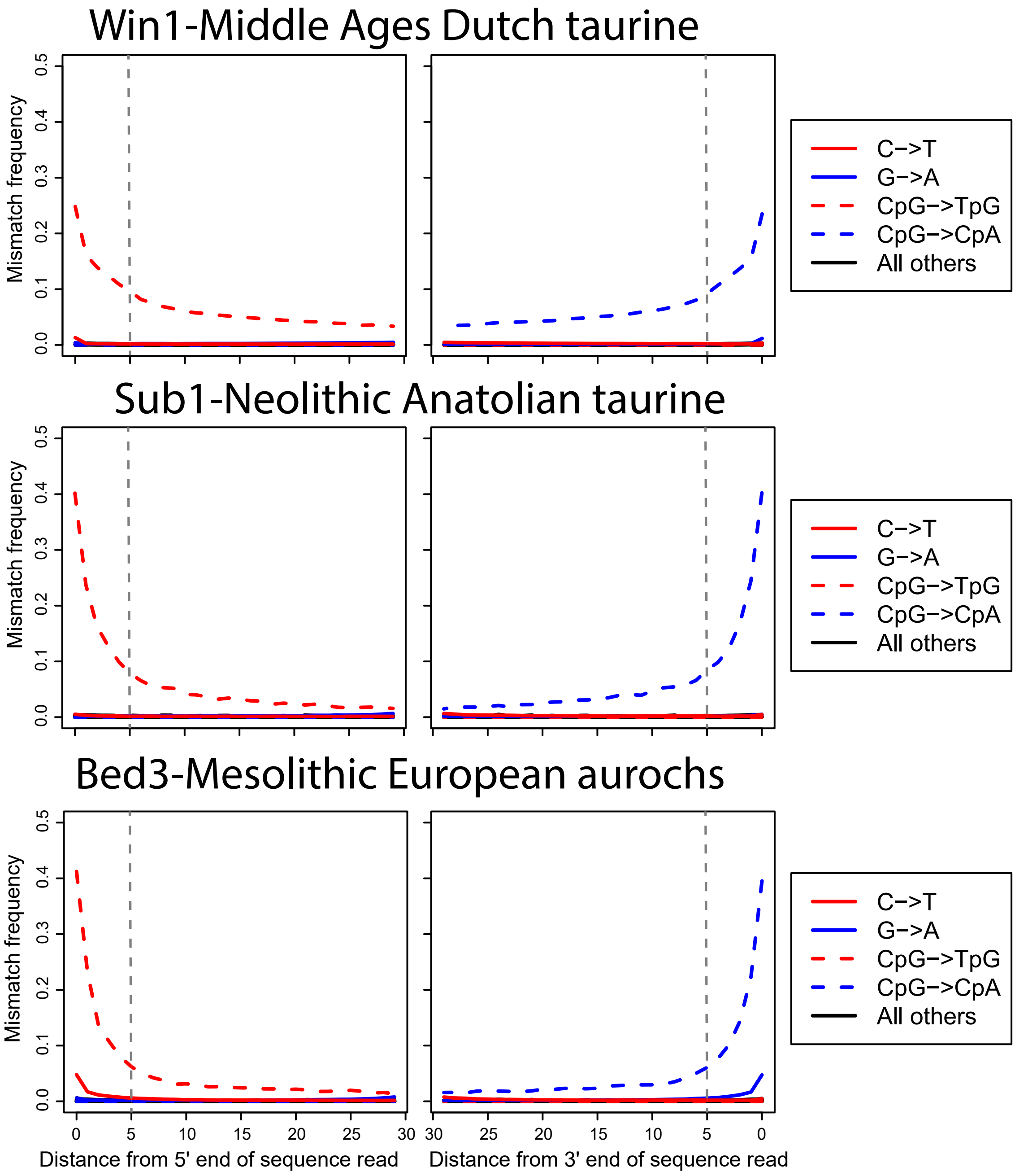

Figure S5. Damage profiles at CpG sites for Win1, Sub1, Bed3 demonstrating the damage patterns typical of ancient DNA. This was calculated prior to the soft-clipping of five base pairs from both ends, clipping is denoted by the black dashed line.
The soft-clipped BAM files were the input for the imputation pipeline. The damage at CpG sites is on average 2.5% higher in Win1 compared to Sub1 and Bed3.

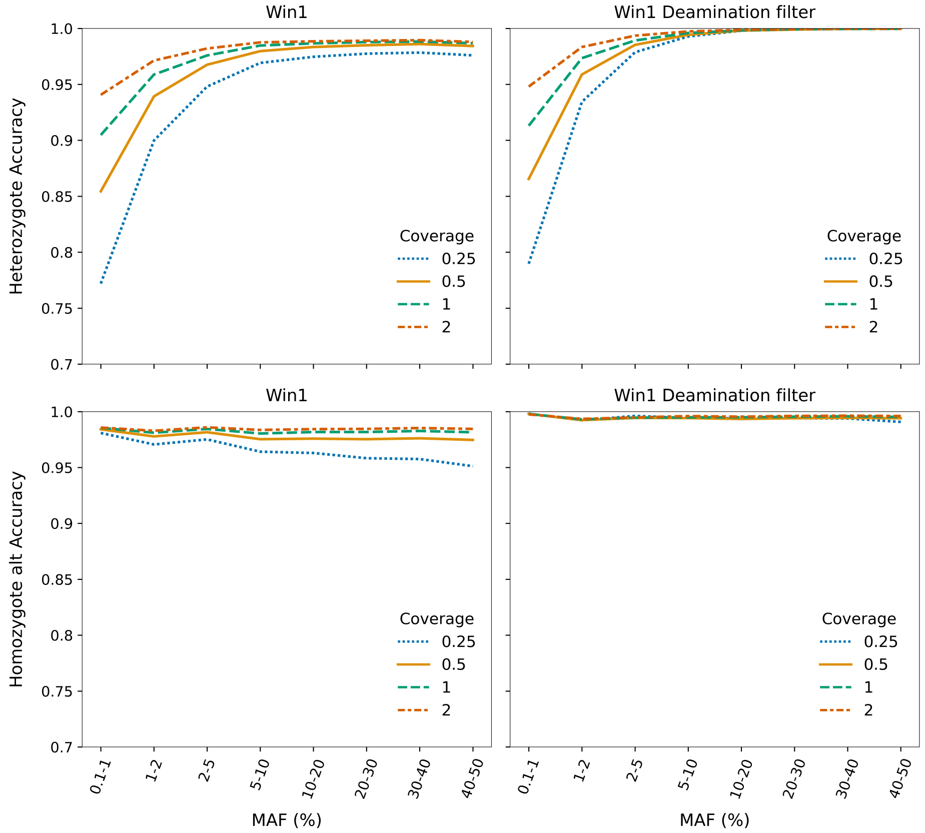

Figure S6. The imputation accuracy for Win1 without a deamination filter and with deamination filter prior to imputation. An improvement in accuracy is shown when Win1 was filtered for deamination.

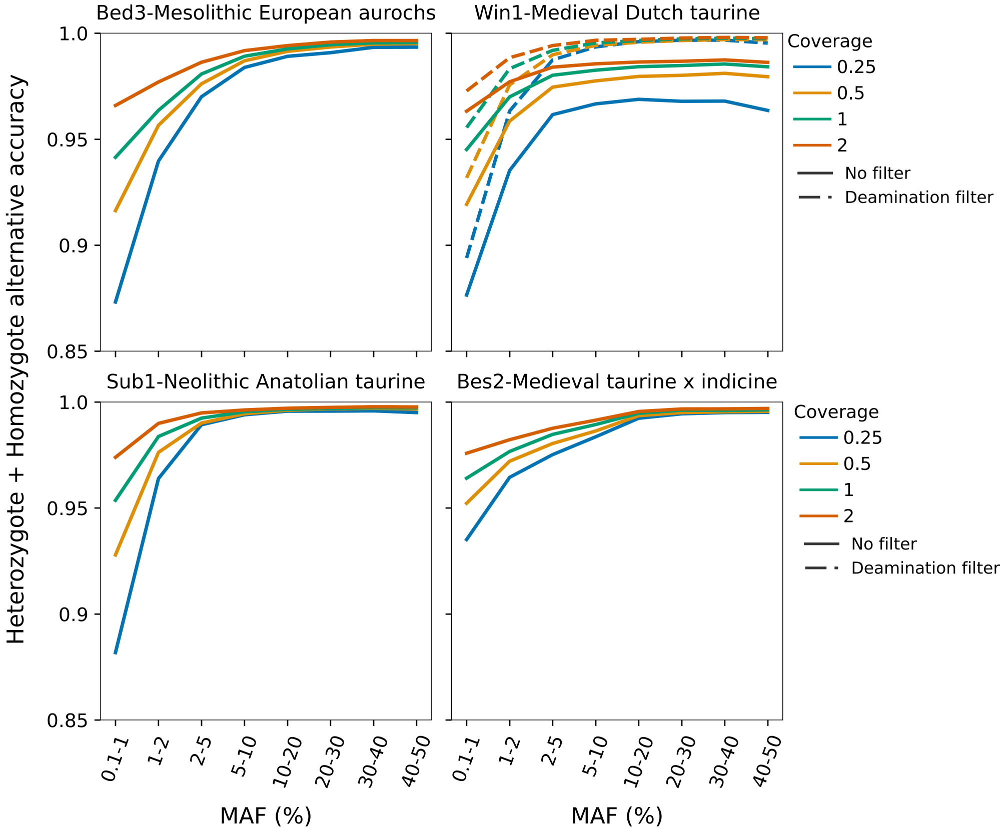

Figure S7. Accuracy of imputation of heterozygote and homozygote alternative sites at different minor allele frequency (MAF) bins for different downsampled coverages and without and with deamination filter prior to imputation, for all sites. Downsampled coverage is denoted by colour and deamination filter is denoted by linestyle.

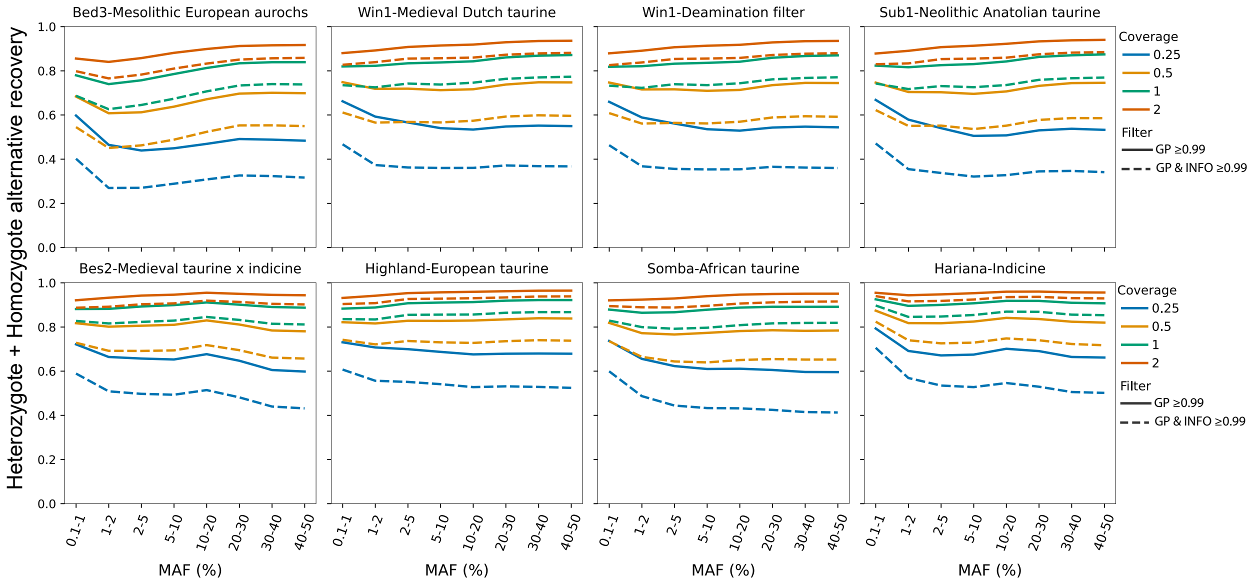

Figure S8. Recovery of imputation of heterozygote and homozygote alternative sites at different minor allele frequency (MAF) bins and different GP and INFO filters. GP & INFO and GP filter are denoted by line style and colour. An additional graph is included for Win1 to demonstrate the effect on the recovery of a deamination filter prior to imputation.

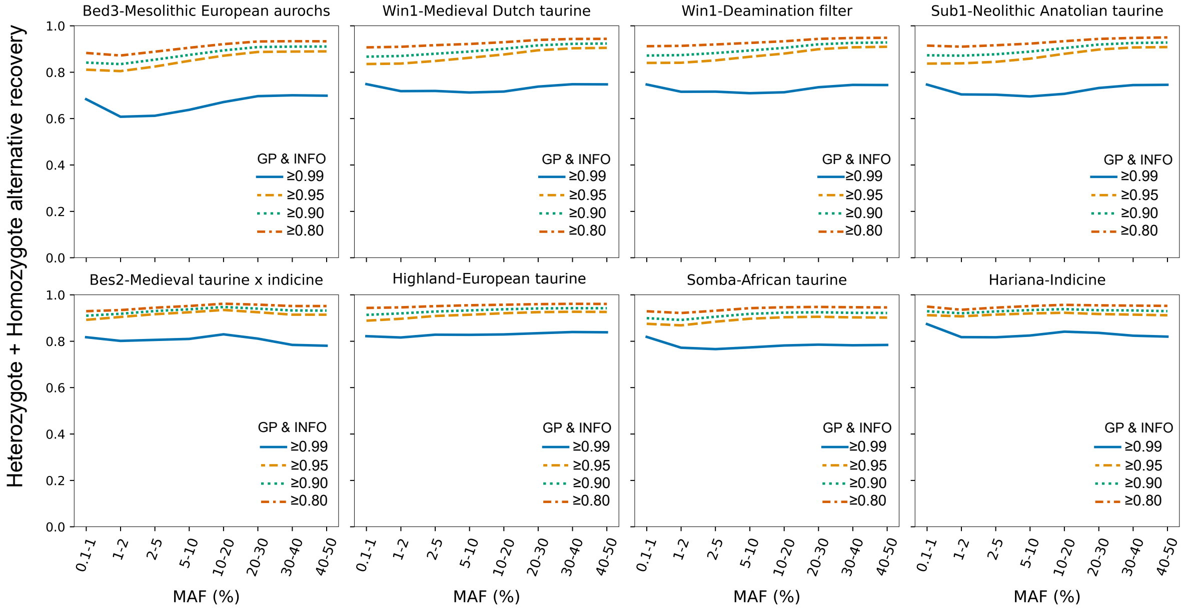

Figure S9. Recovery of imputation of heterozygote and homozygote alternative sites at different minor allele frequency (MAF) bins and different GP and INFO filters, shown for 0.5x coverage for all sites. GP & INFO filter is denoted by line style and colour. An additional graph is included for Win1 to demonstrate the effect on the recovery of a deamination filter prior to imputation.

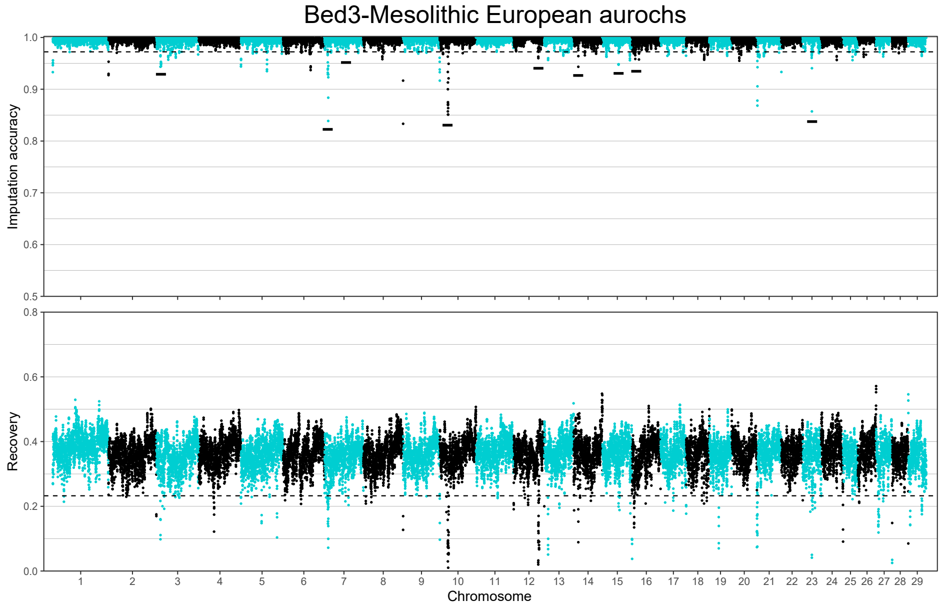

Figure S10. Imputation accuracy and recovery across chromosomes for Bed3, divided in sliding windows. Upper panel shows imputation accuracy, where the dotted line equals the 0.01 percentile. Lower panel shows recovery, where the dotted line equals the 0.01 percentile. Each dot represents a 100kb step-size window. Black bars denote reduced accuracy regions shared across the majority of the test samples.

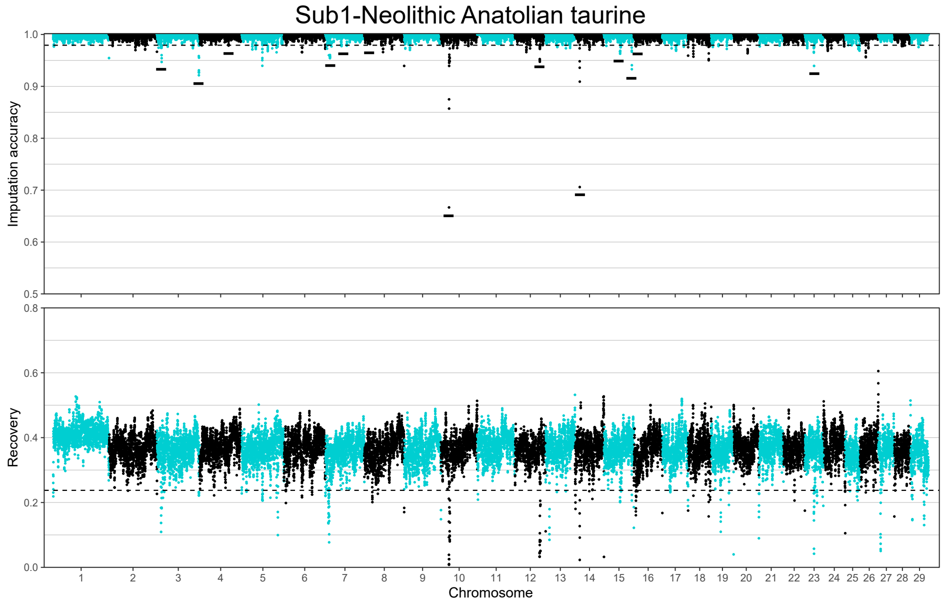

Figure S11. Imputation accuracy and recovery across chromosomes for Sub1, divided in sliding windows. Upper panel shows imputation accuracy, where the dotted line equals the 0.01 percentile. Lower panel shows recovery, where the dotted line equals the 0.01 percentile. Each dot represents a 100kb step-size window. Black bars denote reduced accuracy regions shared across the majority of the test samples.

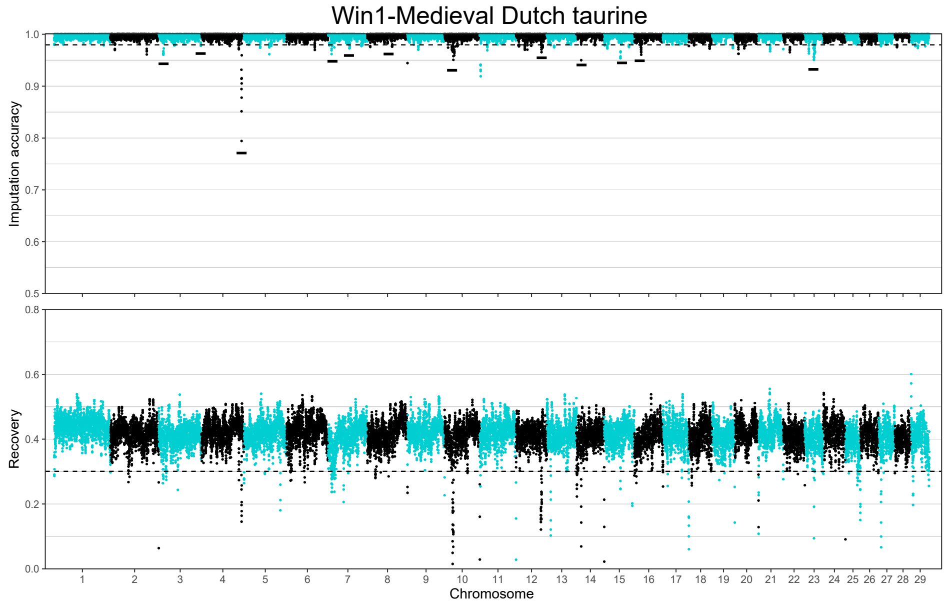

Figure S12. Imputation accuracy and recovery across chromosomes for Win1, divided in sliding windows. Upper panel shows imputation accuracy, where the dotted line equals the 0.01 percentile. Lower panel shows recovery, where the dotted line equals the 0.01 percentile. Each dot represents a 100kb step-size window. Black bars denote reduced accuracy regions shared across the majority of the test samples.

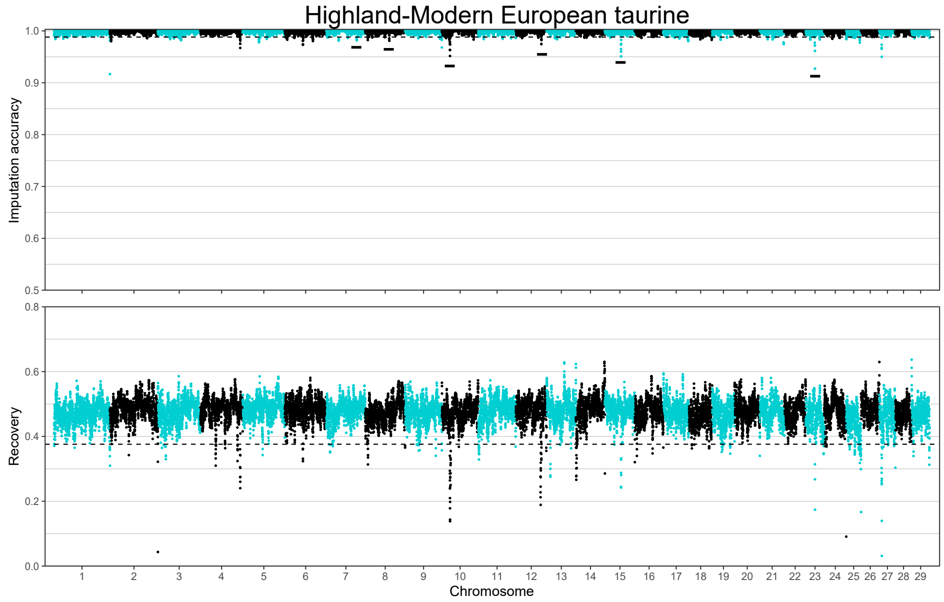

Figure S13. Imputation accuracy and recovery across chromosomes for Highland, divided in sliding windows. Upper panel shows imputation accuracy, where the dotted line equals the 0.01 percentile. Lower panel shows recovery, where the dotted line equals the 0.01 percentile. Each dot represents a 100kb step-size window. Black bars denote reduced accuracy regions shared across the majority of the test samples.

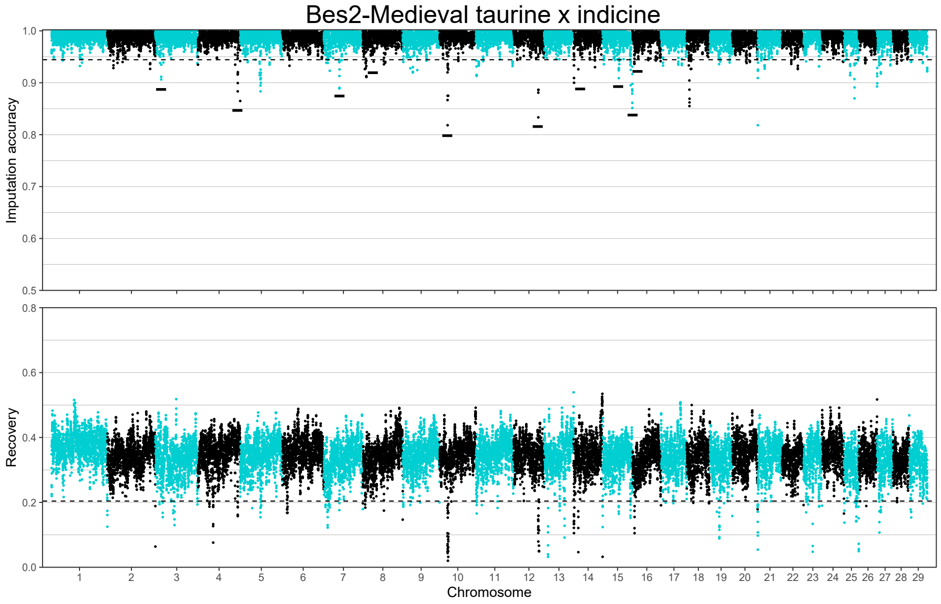

Figure S14. Imputation accuracy and recovery across chromosomes for Bes2, divided in sliding windows. Upper panel shows imputation accuracy, where the dotted line equals the 0.01 percentile. Lower panel shows recovery, where the dotted line equals the 0.01 percentile. Each dot represents a 100kb step-size window. Black bars denote reduced accuracy regions shared across the majority of the test samples.

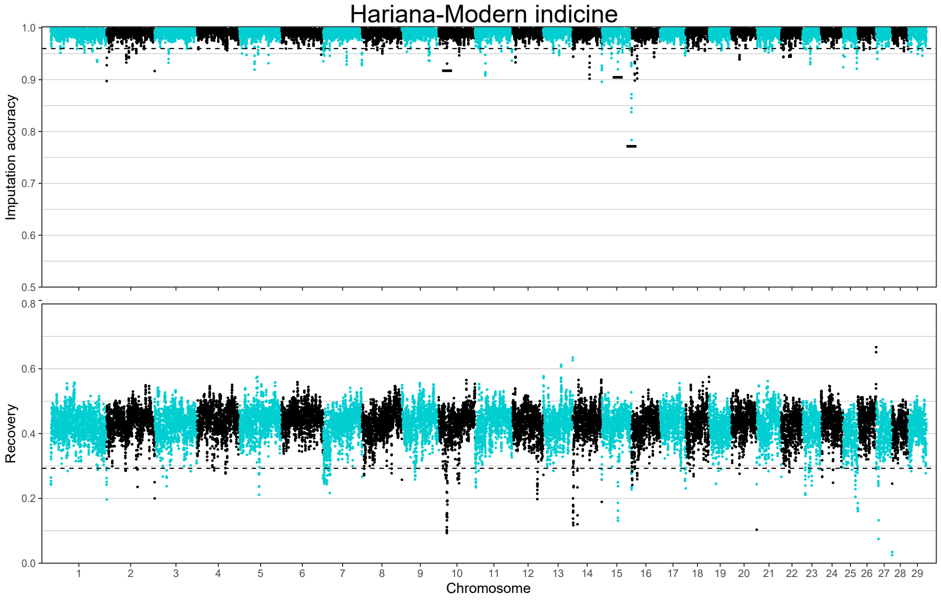

Figure S15. Imputation accuracy and recovery across chromosomes for Hariana, divided in sliding windows. Upper panel shows imputation accuracy, where the dotted line equals the 0.01 percentile. Lower panel shows recovery, where the dotted line equals the 0.01 percentile. Each dot represents a 100kb step-size window. Black bars denote reduced accuracy regions shared across the majority of the test samples.

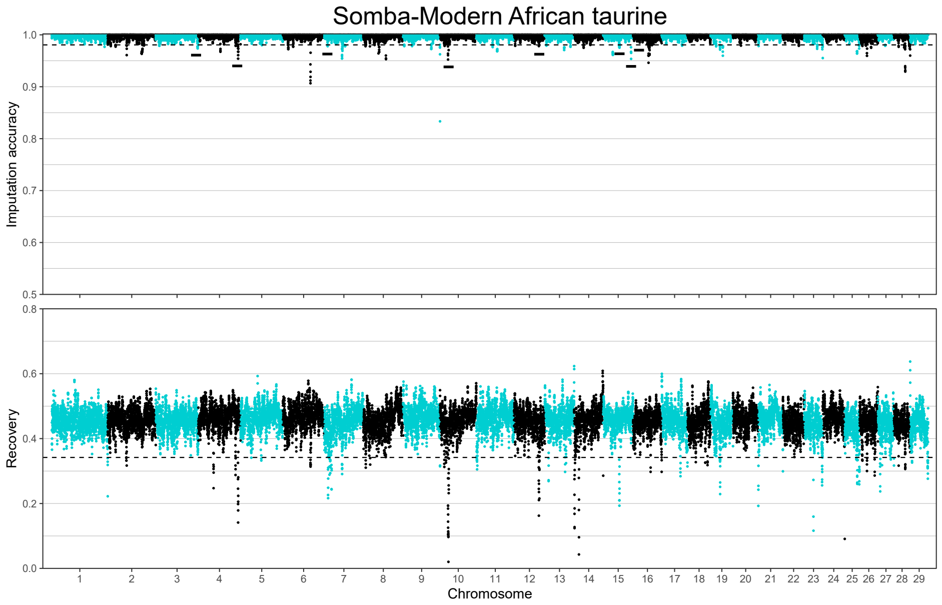

Figure S16. Imputation accuracy and recovery across chromosomes for Somba, divided in sliding windows. Upper panel shows imputation accuracy, where the dotted line equals the 0.01 percentile. Lower panel shows recovery, where the dotted line equals the 0.01 percentile. Each dot represents a 100kb step-size window. Black bars denote reduced accuracy regions shared across the majority of the test samples.

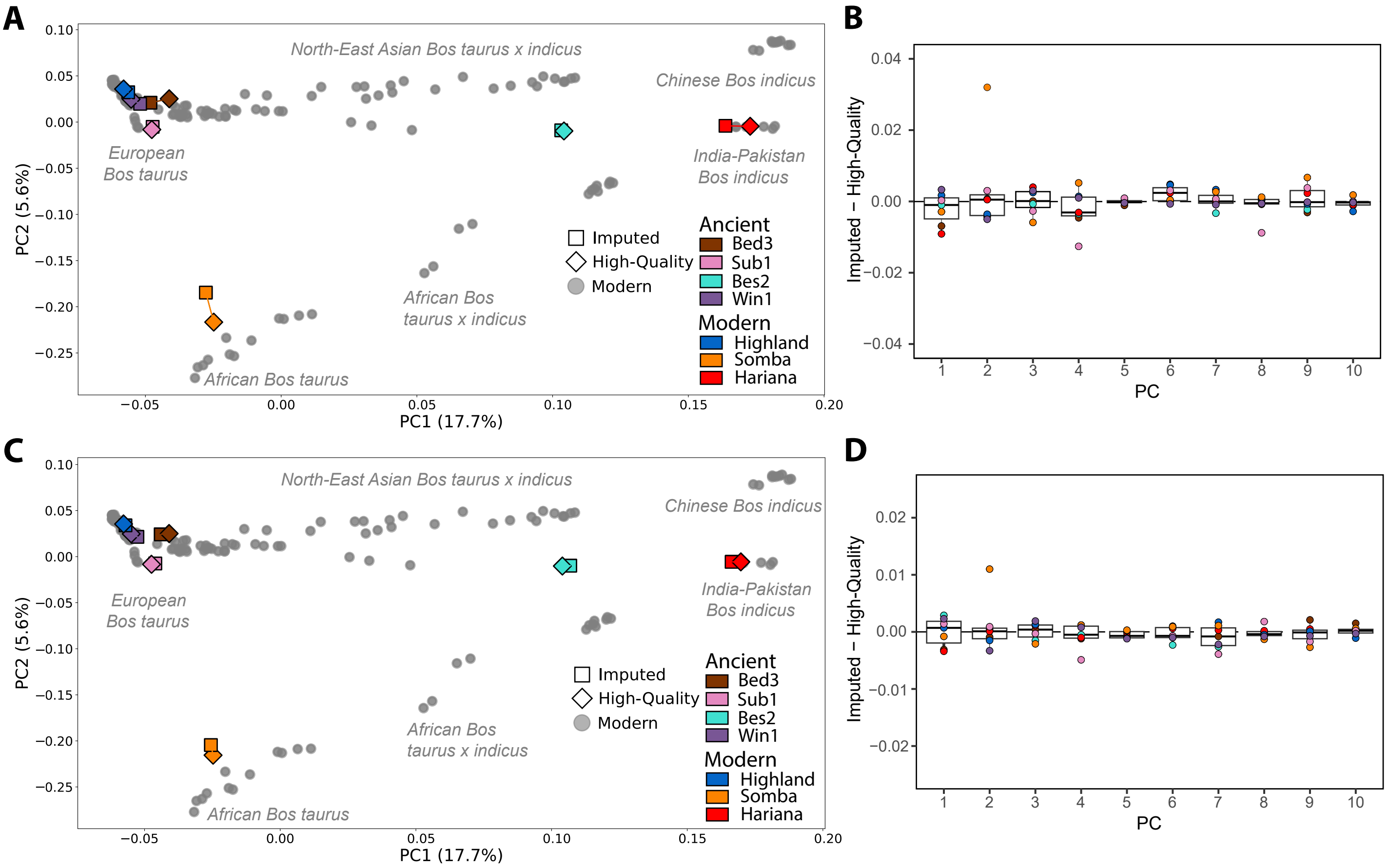

Figure S17. Principal Components Analysis (PCA) of imputed genotypes and high-quality genotypes of the seven test individuals onto the modern cattle reference panel A: Projections for 0.25x imputed and high-quality genotypes of the test samples onto the modern reference panel along the first two eigenvectors. Dataset was filtered for a MAF ≥2.5%, transversions and LD pruning resulting in 800,694 SNPs. Test individuals are denoted by colour with imputed and high-quality represented by squares and diamonds respectively, while the reference panel individuals are plotted as grey circles. **B:** Boxplots of the normalised differences in the coordinates of the high-quality and 0.25x imputed genomes for the first ten principle components. The horizontal lines of the box pot represent the first quartile, median and third quartile, whiskers represent 1.5 times the quartile range.
C: Projections for 1x imputed and high-quality genotypes as in Panel A, with 840,555 SNPs.
D: Boxplots of the normalised differences in the coordinates of the high-quality and 1x imputed genomes for the first ten principle components, as shown in Panel B, with a different scale on the y-axis.

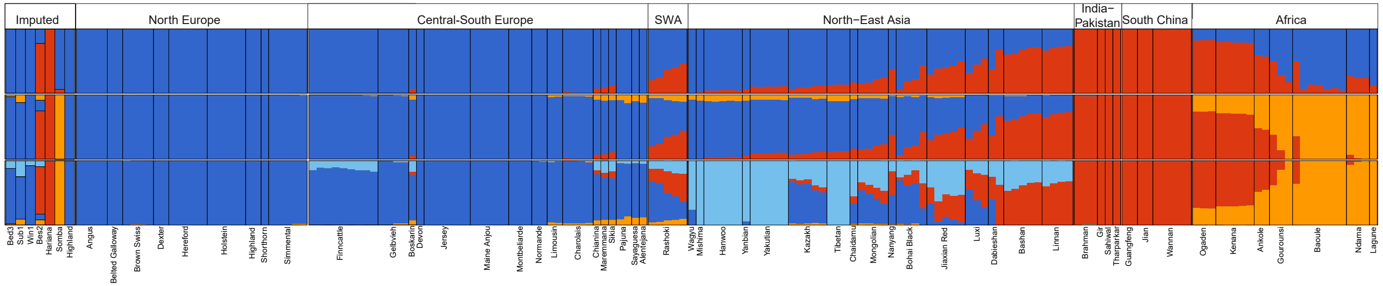

Figure S18. Model based estimates of ancestral coefficients for the seven 0.5x imputed samples and the reference panel for K2-K4. ADMIXTURE analysis based on 800,694 SNPs (MAF ≥2.5%, transversions and LD pruning). Ancestral components are shown for taurine (dark blue), indicine (red), African taurine (orange) and North-East Asian taurine (light blue).
K3-4 had the lowest CV score, and are the optimal K.

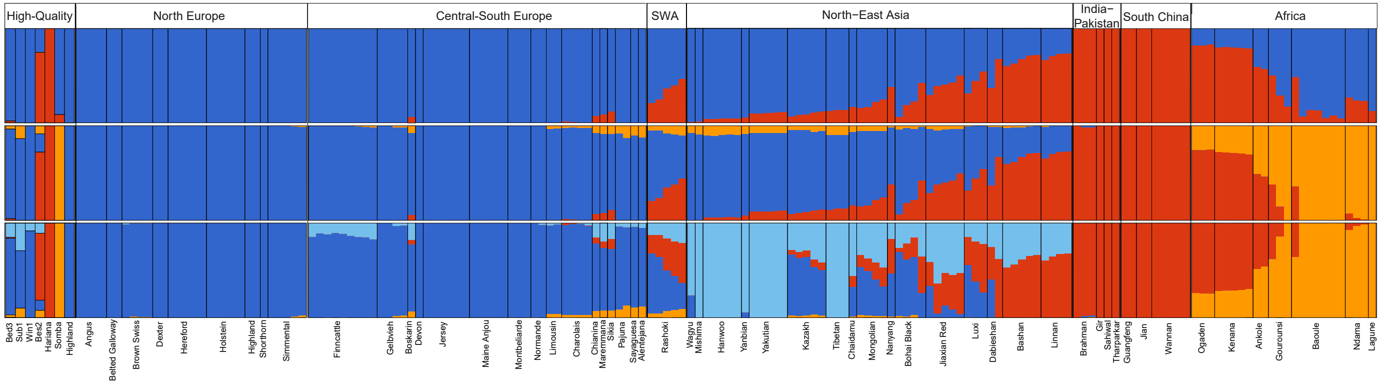

Figure S19. Model based estimates of ancestral coefficients for the seven high-quality genotypes and the reference panel for K2-K4. ADMIXTURE analysis based on 800,694 SNPs (MAF ≥2.5%, transversions and LD pruning). Ancestral components are shown for taurine (dark blue), indicine (red), African taurine (orange) and North-East Asian taurine (light blue).
K3-4 had the lowest CV score, and are the optimal K.

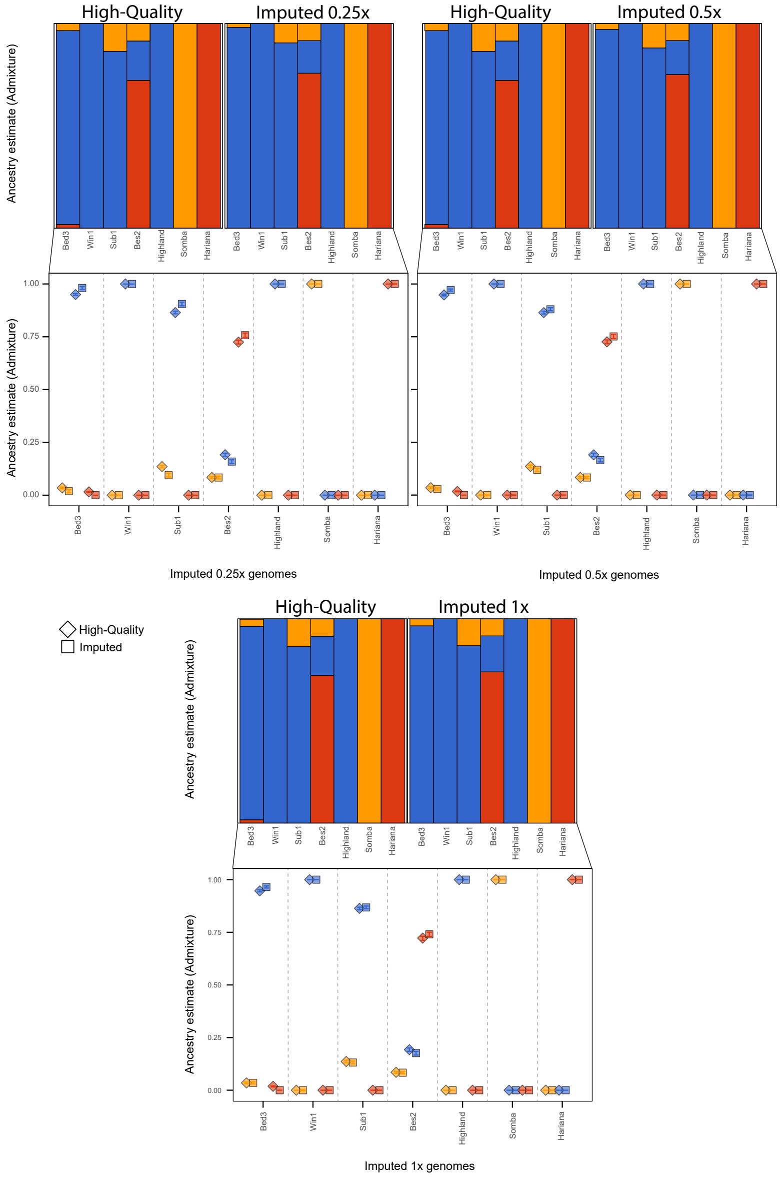

Figure S20. Comparison of the model based estimates of ancestral coefficients for the high quality genotypes and imputed genotypes for K=3. ADMIXTURE analysis based on 800,694 SNPs (MAF ≥2.5%, transversions and LD pruning) run with the reference panel and the seven test samples, only the test samples are plotted for ease. Ancestral components for K=3 are displayed for the 0.25x, 0.5x and 1x imputed (squares) and high-quality validation (diamonds) genotypes of the seven samples, colours are denoted as taurine (dark blue), indicine (red) and African taurine (orange). Error bars represent the deviation from the mean measured as standard errors, calculated by bootstrapping 1000 times.

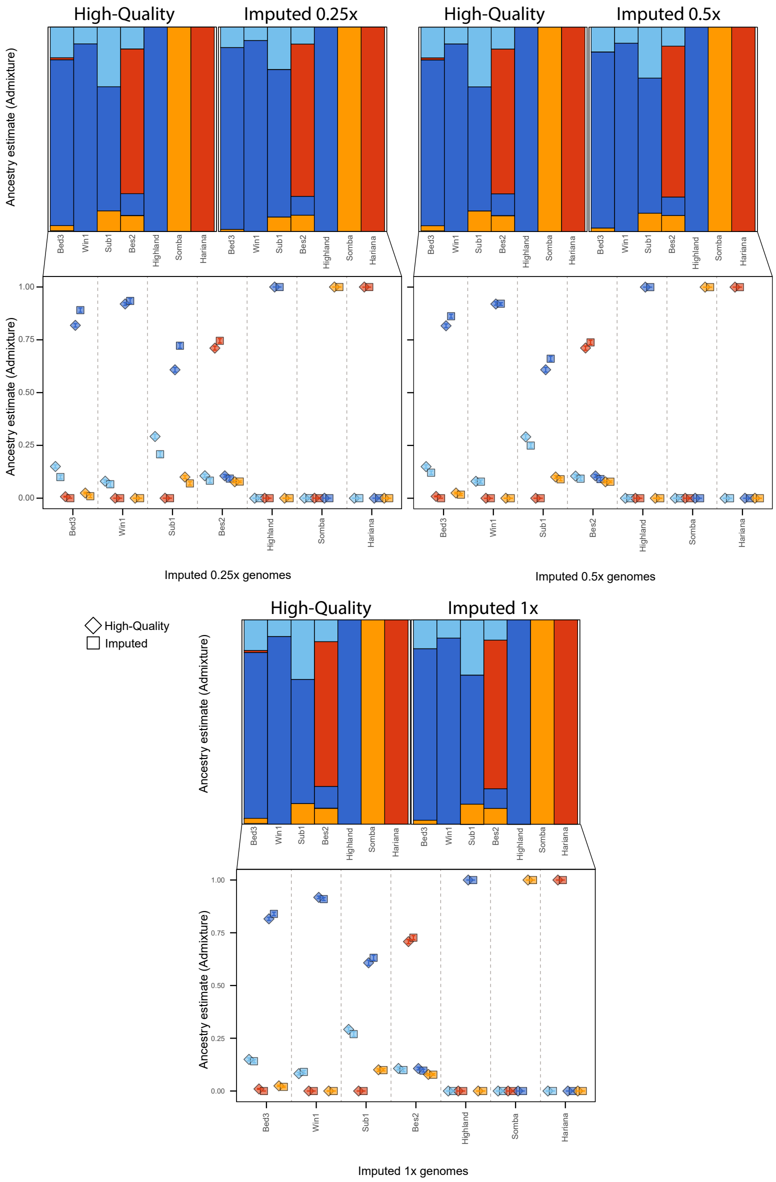

Figure S21. Comparison of the model based estimates of ancestral coefficients for the high quality genotypes and imputed genotypes for K=4. ADMIXTURE analysis based on 800,694 SNPs (MAF ≥2.5%, transversions and LD pruning) run with the reference panel and the seven test samples, only the test samples are plotted for ease. Ancestral components for K=4 are displayed for the 0.25x, 0.5x and 1x imputed (squares) and high-quality validation (diamonds) genotypes of the seven samples, colours are denoted as taurine (dark blue), indicine (red), African taurine (orange) and North-East Asian taurine (light blue). Error bars represent the deviation from the mean measured as standard errors, calculated by bootstrapping 1000 times.

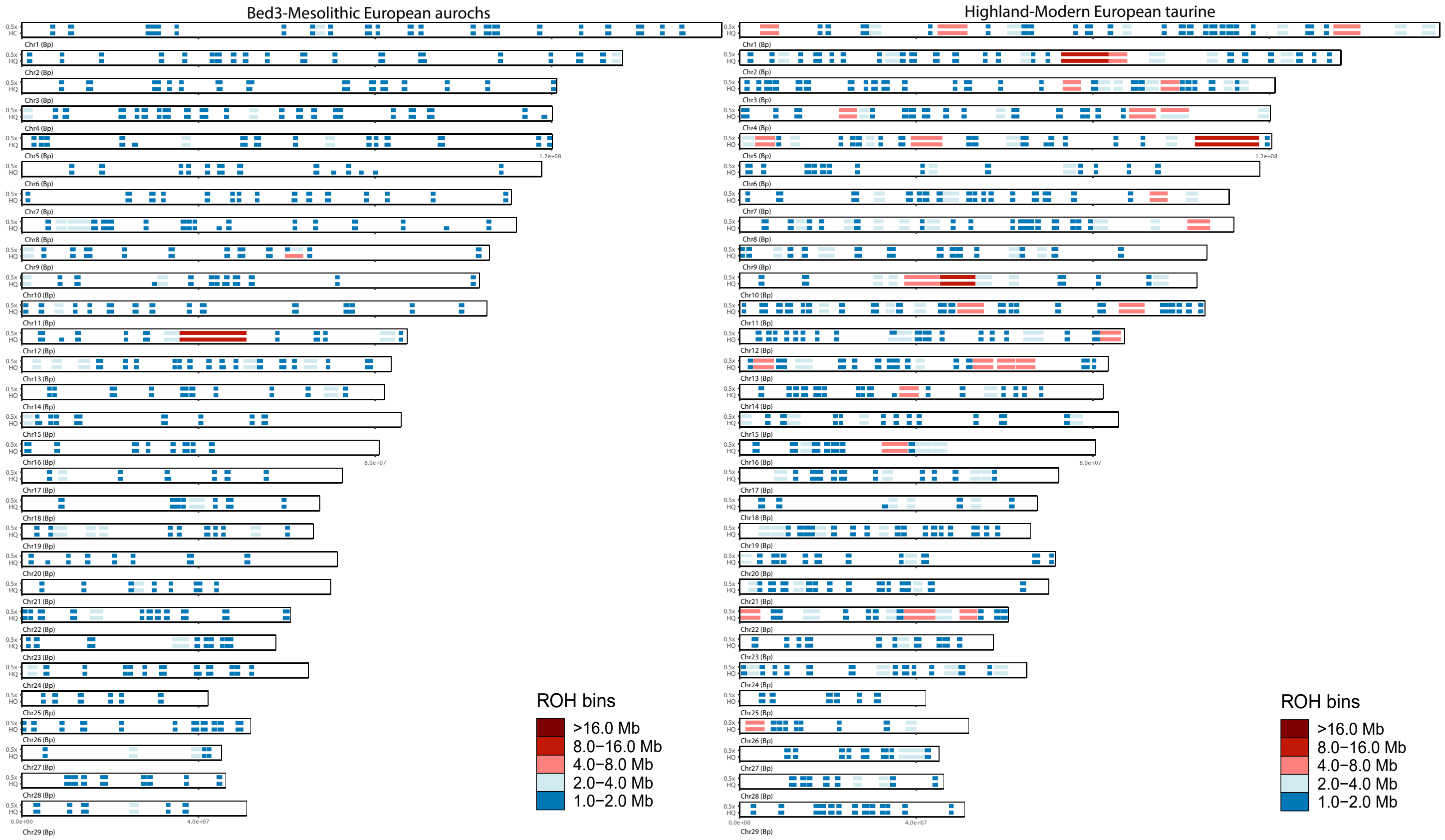

Figure S22. Runs of homozygosity along all chromosomes for Bed3 and the modern European taurine Highland breed for high-quality genotypes and imputed genotypes from the 0.5x downsample. The number of sites used for analysis = 481,786 filtered for a MAF ≥ 2.5%, transversions only and no missingness.

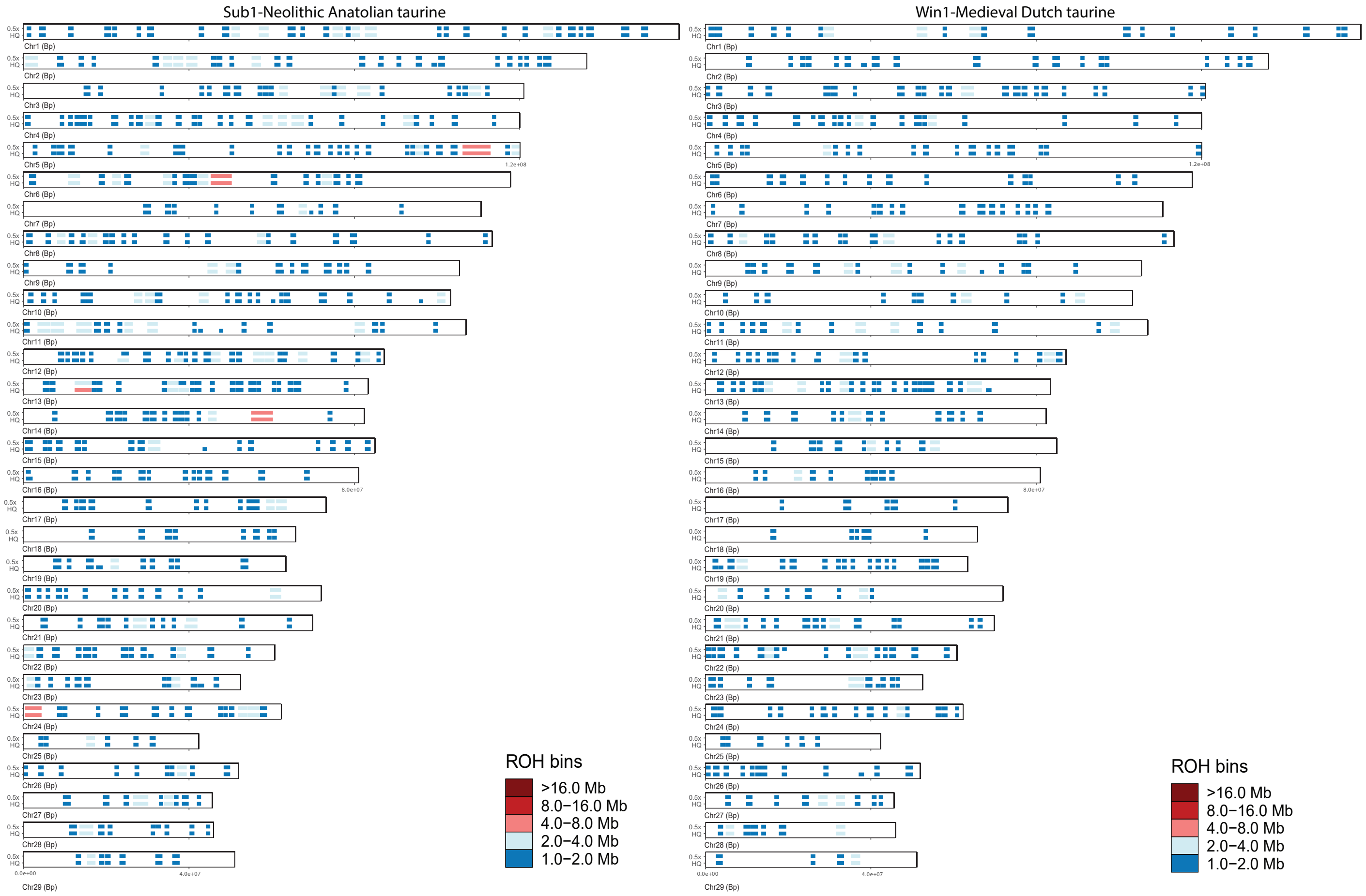

Figure S23. Runs of homozygosity along all chromosomes for Sub1 and Win for high-quality genotypes and imputed genotypes from the 0.5x downsample.
The number of sites used for analysis = 481,786 filtered for a MAF ≥ 2.5%, transversions only and no missingness.

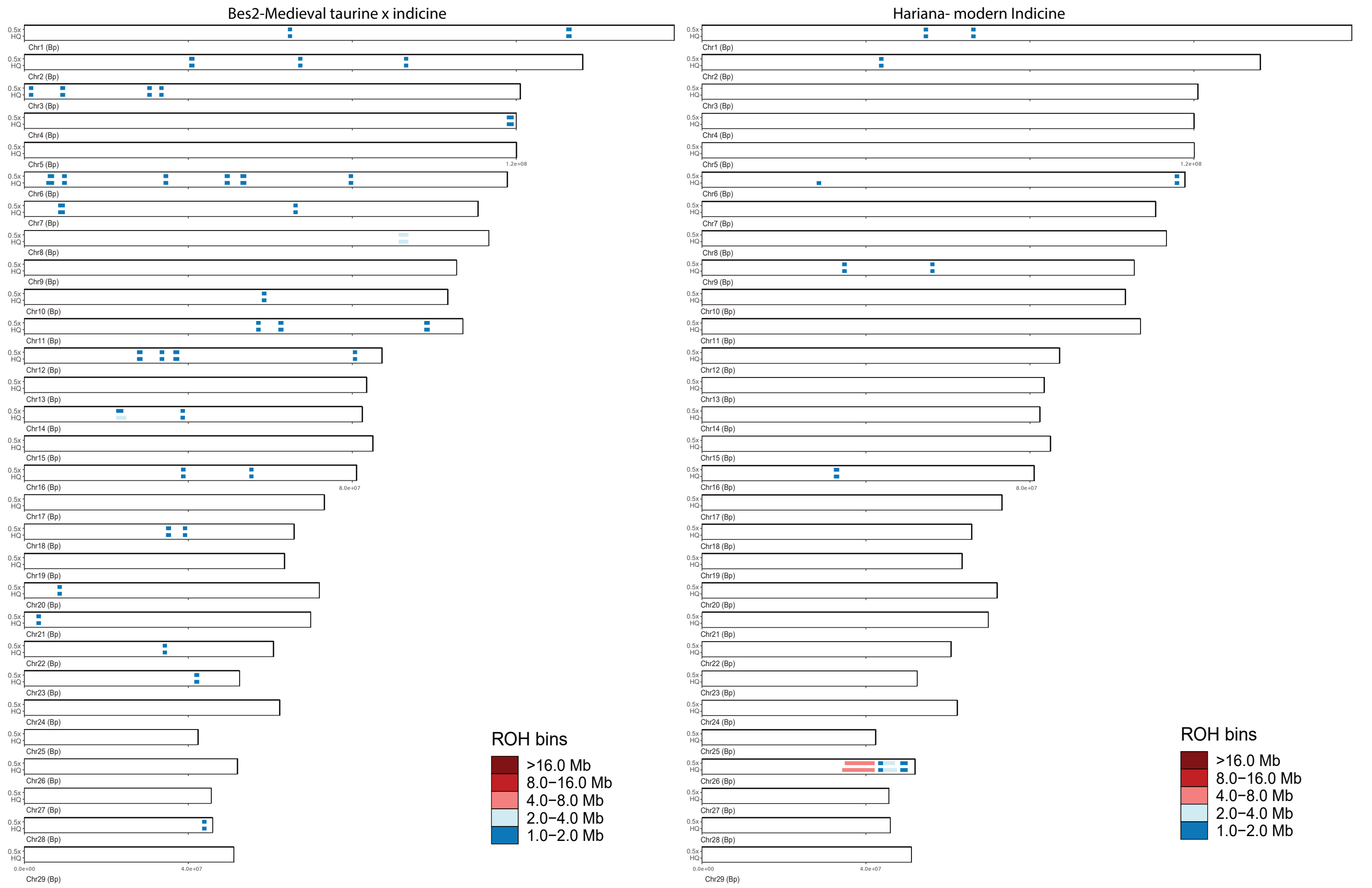

Figure S24. Runs of homozygosity along all chromosomes for Bes2 and the modern indicine Hariana breed for high-quality genotypes and imputed genotypes from the 0.5x downsample. The number of sites used for analysis = 481,786 filtered for a MAF ≥ 2.5%, transversions only and no missingness.

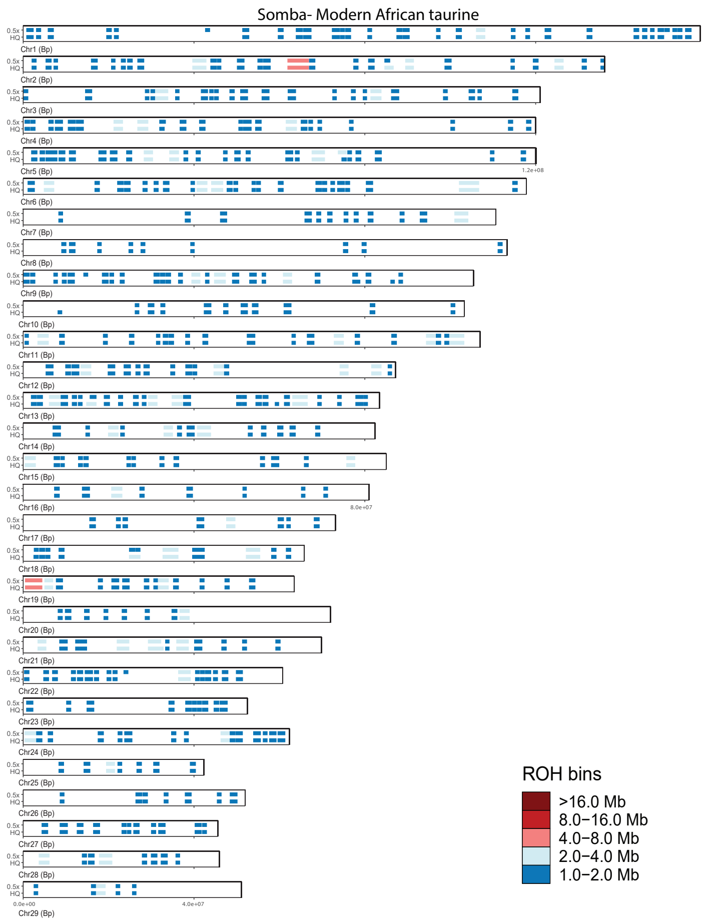

Figure S25. Runs of homozygosity along all chromosomes for the modern African taurine Somba breed for high-quality genotypes and imputed genotypes from the 0.5x downsample.
The number of sites used for analysis = 481,786 filtered for a MAF ≥ 2.5%, transversions only and no missingness.

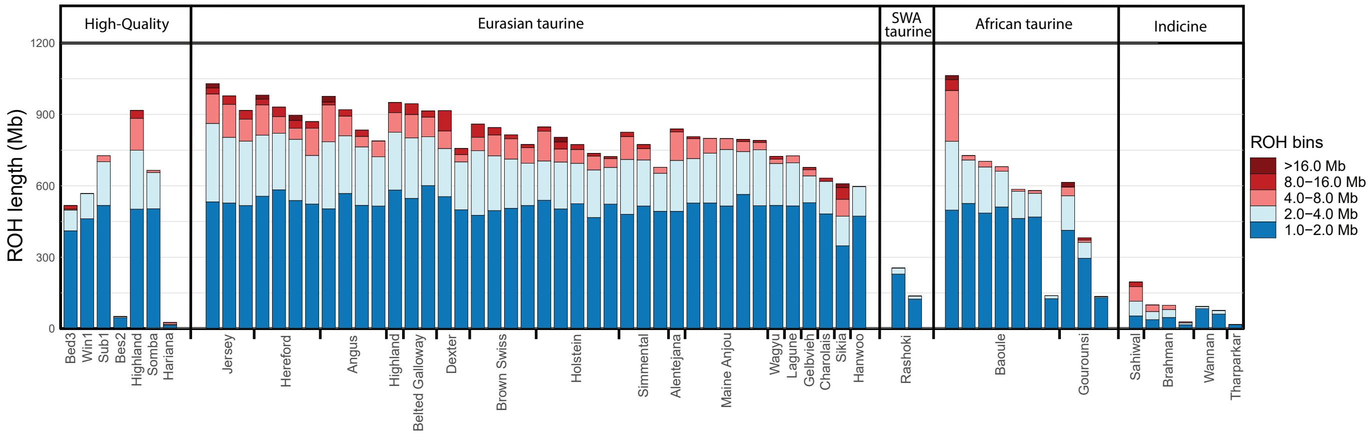

Figure S26. Runs of Homozygosity (ROH) estimates for the high-quality genotypes and the modern reference panel. The total length of ROH split into length bins for the seven test samples and a subset of the modern reference panel (N=60). The number of sites used for analysis = 481,786 filtered for a MAF ≥2.5%, transversions only and no missingness.

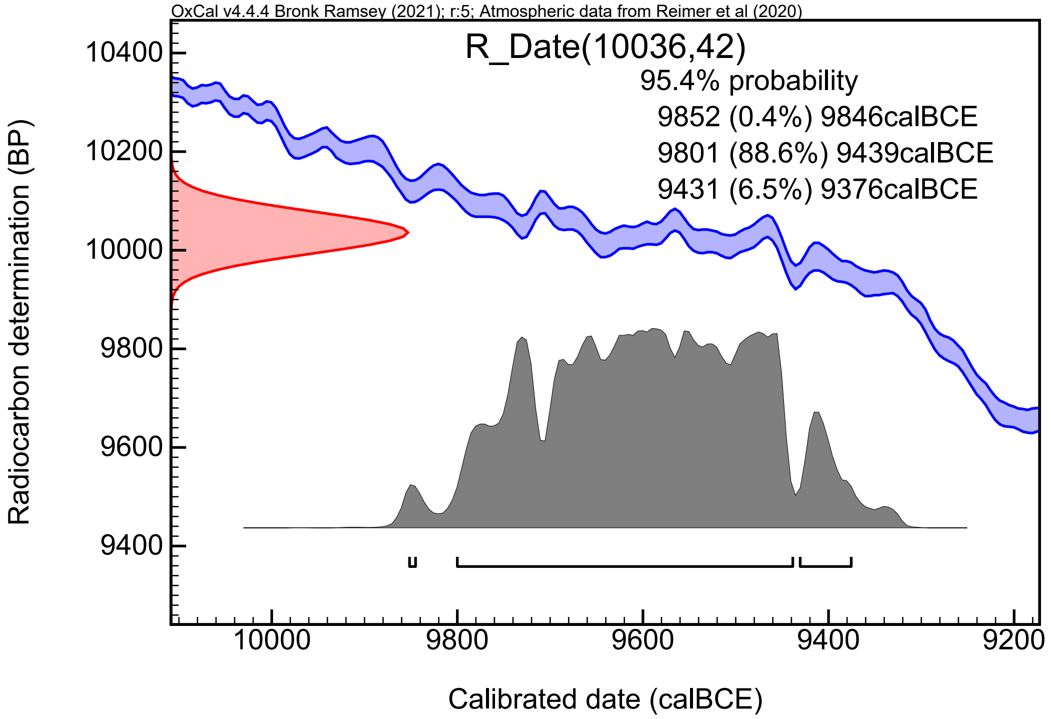

Figure S27. Radiocarbon calibration curve for Bed3

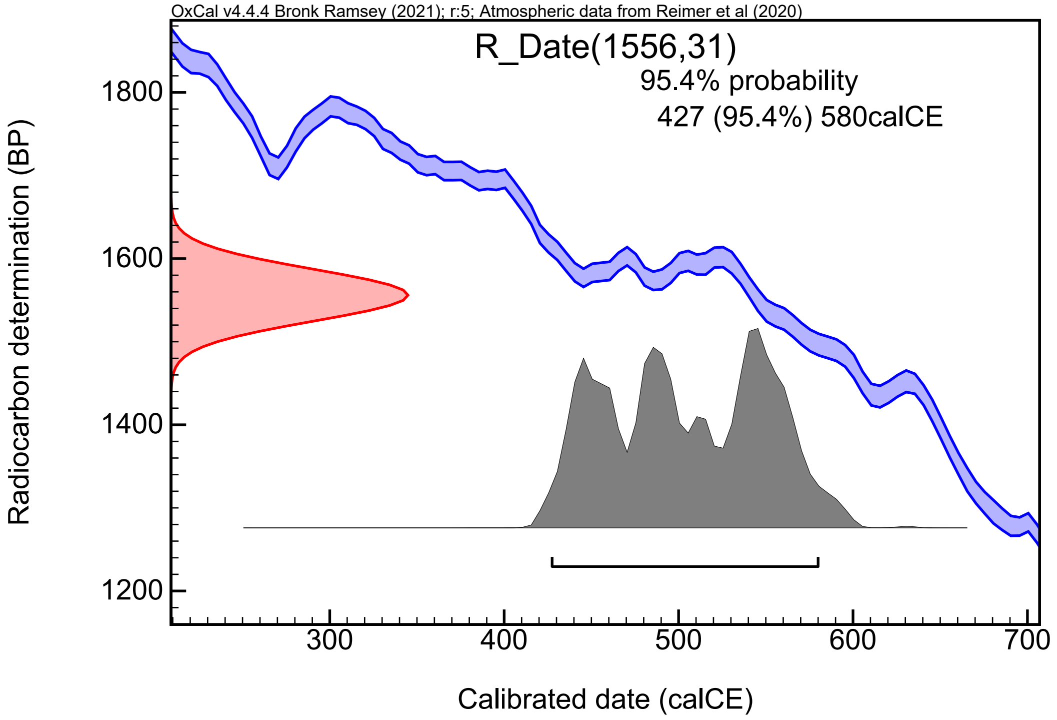

Figure S28. Radiocarbon calibration curve for Win1
